# Synthesis and Evaluation of 5-(Heteroarylmethylene)hydantoins as Glycogen Synthase Kinase-3β Inhibitors

**DOI:** 10.3390/ph17050570

**Published:** 2024-04-29

**Authors:** Nicholas O. Schneider, Kendra Gilreath, Daniel J. Burkett, Martin St. Maurice, William A. Donaldson

**Affiliations:** 1 Department of Biological Sciences, Marquette University, P.O. Box 1881, Milwaukee, WI 53201, USA; 2 Department of Chemistry, Marquette University, P.O. Box 1881, Milwaukee, WI 53201, USA

**Keywords:** nitrogen heterocycles, glycogen synthase kinase 3β, computational docking

## Abstract

Glycogen synthase kinase-3 (GSK-3) is a serine/threonine kinase which plays a center role in the phosphorylation of a wide variety of proteins, generally leading to their inactivation. As such, GSK-3 is viewed as a therapeutic target. An ever-increasing number of small organic molecule inhibitors of GSK-3 have been reported. Phenylmethylene hydantoins are known to exhibit a wide range of inhibitory activities including for GSK-3β. A family of fourteen 2-heterocycle substituted methylene hydantoins (**14**, **17**–**29**) were prepared and evaluated for the inhibition of GSK-3β at 25 μM. The IC_50_ values of five of these compounds was determined; the two best inhibitors are 5-[(4′-chloro-2-pyridinyl)methylene]hydantoin (IC_50_ = 2.14 ± 0.18 μM) and 5-[(6′-bromo-2-pyridinyl)methylene]hydantoin (IC_50_ = 3.39 ± 0.16 μM). The computational docking of the compounds with GSK-3β (pdb 1q41) revealed poses with hydrogen bonding to the backbone at Val135. The 5-[(heteroaryl)methylene]hydantoins did not strongly inhibit other metalloenzymes, demonstrating poor inhibitory activity against matrix metalloproteinase-12 at 25 μM and against human carbonic anhydrase at 200 μM, and were not inhibitors for *Staphylococcus aureus* pyruvate carboxylase at concentrations >1000 μM.

## 1. Introduction

Glycogen synthase kinase 3 (GSK-3) is a serine/threonine kinase which was originally found to phosphorylate and inhibit glycogen synthase, an enzyme responsible for glycogen synthesis [1]. GSK-3 is phosphorylated on tyrosine and is active in resting cells [2]. GSK-3 was identified as phosphorylating over 100 different proteins and it has been said that it “may be the busiest kinase in most cells” [3]. Phosphorylation by GSK-3 generally leads to the inactivation of the phosphorylated protein [4]. There are two isoforms, GSK-3α and GSK-3β, which have high homology in the region containing motifs common to all protein kinases, while the N- and C-terminal regions have considerably less homology [5]. Since GSK-3β affects multiple signaling pathways, it has become a therapeutic target for type-2 diabetes [6], psychiatric disorders [7,8,9,10], cancer [11,12,13], Alzheimer’s disease [14,15,16], and even SARS-CoV-2 [17]. 

**Figure 1:**
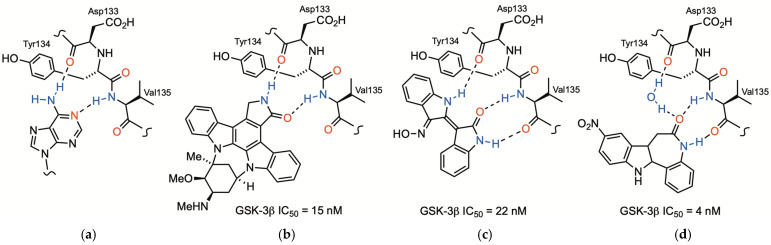
Representations of the interactions of the adenine ring of non-hydrolysable AMP-PNP (**a**), and the potent GSK-3β inhibitors staurosporine (**b**), indirubin-3-monoxime (**c**), and alsterpaullone (**d**) in the ATP binding domain of GSK-3β as revealed in X-ray structures (Ref. [19]). Other hydrogen-bonding interactions, not common among these co-crystal structures, are omitted.

The structure of GSK-3β consists of an N-terminal β-strand domain and a C-terminal α-helix domain joined by a glycine-rich loop and hinge [18]. The ATP-binding domain is at the interface of the β-strand and α-helix domains. The X-ray crystal structures of human GSK-3β with AMP-PNP, and the inhibitors staurosporine, indirubin-3-monoxime, and alsterpaullone reveal important interactions with the backbone carbonyl of Asp133 and the backbone amide NH and/or the carbonyl of Val135 (Figure 1) [19]. An ever-growing number of small organic molecule GSK-3β inhibitors have been reported, with additional crystal structures or docking studies reinforcing these crucial interactions [20]. 

**Figure 2:**
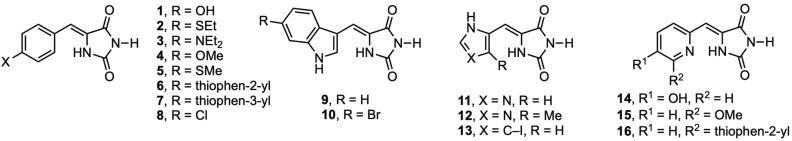
Known phenylmethylenehydantoins and 5-(heteroarylmethylene)hydantoins.

5-(4-Hydroxybenzylidene)hydantoin (**1**, Figure 2) [21], 3′-deimino-2′,4′-bis(dimethyl)-3′-oxoaplysinopsin (**9**) [22], and the 6-bromo analog (**10**) [21,22] are naturally occurring substituted 5-methylidenehydantoins isolated from marine invertebrates. Compound **1** and several synthetic analogs (**2**–**8**) are reported to exhibit GSK-3β inhibitory activity in the range 4–18 μM [23]. Several other 5-(heteroarylmethylene)hydantoins have been evaluated as cancer migration inhibitors (**11**–**16**, Figure 2), but only compound **16** exhibited notable activity [24].

**Scheme 1:**
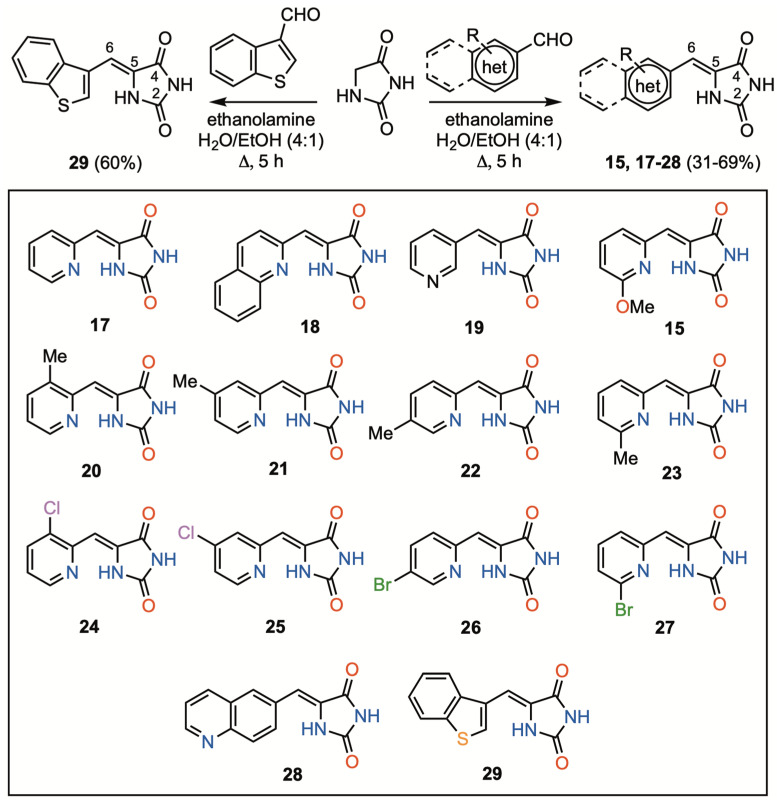
Synthesis of 5-(heteroarylmethylene)hydantions.

Inspired by the GSK-3β inhibitory activity exhibited by compounds **1**–**8** [23], we prepared a series of 5-(heteroarylmethylene)hydantoins (**15**, **17**–**29**, Scheme 1) and examined their inhibitory activity against GSK-3β. Intramolecular hydrogen bonding between the N(1)–H bond of the hydantoin and the nitrogen of a (2-pyridinyl) substituent was anticipated to impart some conformational bias about the C(6) to aryl bond [25], and thus substituents at positions C3, C4, and C6 of the pyridine ring would have directional orientation within the binding pocket of GSK-3β. To this end, results from compounds **15**, **18**, and **20**–**23** would illuminate any steric constraints within the binding pocket. Results from compounds **24**–**28** might provide information about the hydrogen bonding acceptor ability of the halogen atoms within these structures, the potential for which is underexplored in compounds **1**–**8** [23]. Compound **28** was chosen as an isomer of **18** in which the hydantoin is attached at a different position of the quinoline ring. Since 5-(pyridine-2-ylmethylene)hydantoin (**17**) is known to form chelated complexes with metals [26], we desired to evaluate the possibility that these compounds might non-specifically inhibit metal-dependent enzymes. Our group’s previous experience with evaluating 3-aryl-2-hydroxypropenoic acids as inhibitors for *Staphylococcus aureus* pyruvate carboxylase (*Sa*PC) [27], including selectivity against matrix metalloproteinase-12 (MMP-12) and human carbonic anhydrase (*h*CAII) (two proteins in a well-established metalloprotease panel [27]), made these a logical choice for the selectivity studies included here too.

## 2. Results and Discussion

### 2.1. Chemistry

Four known (**15** and **17**–**19**) and ten new 5-(heteroarylmethylene)hydantoins (**20**–**29**) were prepare through the Knovenegel condensation of hydantoin with heteroarylaldhydes (Scheme 1). In most cases, the product precipitated from the ethanol/water reaction mixture; however, in the case of **21**, **23**, **24**, and **25**, several drops of conc. HCl were added to induce precipitation. Products **15** and **17**–**19** were identified by means of a comparison of their physical and/or spectral data to values from the literature [24,25,28]. The structures of 6-substituted-5-methylenehydantoins **20**–**29**, were assigned on the basis of their NMR spectral data. In particular, singlets in their ^1^H NMR spectra in the range of *δ* 6.39–6.48 ppm and signals in their ^13^C NMR spectra in the range *δ* 165 and 101–105 ppm correspond to H6, C2, and C6, respectively [29]. The exception to this is **24**, where the signal for H6 appears at *δ* 6.61 ppm. This additional downfield shift is due to the proximity of the 3-chloro substituent. 

### 2.2. Biological Evaluation

#### 2.2.1. Inhibition of GSK-3β

The ability of 5-(heteroarylmethylene)hydantoins **15** and **17**–**29** to inhibit GSK-3β at 25 μM was measured in a kinase HotSpot^TM^ assay with 10 μM ATP; measurements of % activities were made in duplicate and values are reported as the average (Table 1). All compounds exhibited some inhibitory activity at 25 μM. The substituted 5-(pyridin-2-yl)methylenehydantoins **15**, **23**, **25**, and **27**, and the 5-(quinoline-6′-yl)methylenehydantoin **28** were further assessed in 10-point inhibition measurements conducted in triplicate (Table 1). All five were single-digit micromolar inhibitors, with the two most potent ones being **25** and **27** (IC_50_ = 2.14 ± 0.18 μM and 3.39 ± 0.16 μM, respectively). While the GSK-3β inhibitory activities of these compounds are considerably less potent than the highly potent inhibitors stauroporine (IC_50_ = 15 nM), indirubin-3-monoxime (IC_50_ = 22 nM), and alsterpaullone (IC_50_ = 4 nM), they are nonetheless in the same range previously reported for substituted 5-phenylmethylenehydantoins (**1**–**8**) [23]. It can be concluded that the presence of a nitrogen in the aryl ring attached to the 5-methylenehydantoin does not improve, or diminish, the activity of these compounds.

#### 2.2.2. Molecular Docking

**Figure 3:**
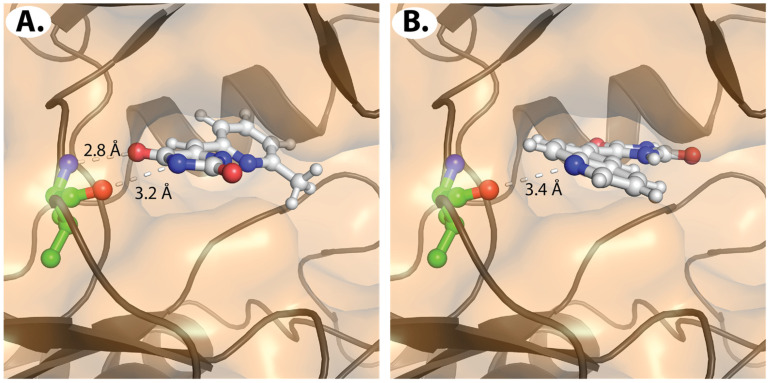
(**A**) Docked structure of (*Z*)-5-[(6′-methyl-2-pyridinyl)methylene]-2,4-imidazolidinedione (**23**) in the binding pocket of GSK3-β (pdb code 1q41) with H-bonding between N(3)–H and C(4)=O with the amide linkage of Val. (**B**) Docked structure of (*Z*)-5-[(6′-quinolinyl)methylene]-2,4-imidazolidinedione (**28**) in the binding pocket of GSK3-β.

The docking of **15** and **17**–**29** into the ATP binding cleft of GSK-3β (pdb 1q41 [19]) was calculated with the Mcule online one-click program which uses the Vina docking algorithm (https://mcule.com, accessed on 8 February 2024). Vina uses a sophisticated gradient optimization method in its local optimizing procedure, and was designed to improve the speed of the execution and accuracy [30]. The authors of this program describe the scoring function is ‘more of a “machine learning” than directly physics-based in its nature’. Structures **15**, **17**–**27**, and **29** docked with modest affinity (–6.7 to –7.8 kcal/mol), where the best scored pose indicated hydrogen bonding interactions between the backbone carbonyl C=O and amide N–H of Val135 with the N–H at position 3 and C=O at position 4 of the hydantoin ring, respectively, as represented by the best docking pose for **23** (Figure 3A). Notably, there was no discernable hydrogen bonding to the backbone N–H of Asp133. In contrast, while the computational docking of **28** produced the best score, the top 4 poses reversed the orientation of the molecule such that the hydantoin ring is buried deep in the binding pocket where it does not make any interactions with the backbone amide group of Val135 (Figure 3B). 

The predictive value of these computations may be limited. Indirubin-3-monoxime is the ligand in PDB 1q41. The top four docking results from the online Mcule program for indirubin-3-monoxime have the oxime OH oriented toward Val 135. This may be due to parameters which overemphasize the energy of this interaction. Notably, the docking of the methyl ether of indirubin-3-oxime resulted in an orientation of the ligand more closely matching the crystal structure. 

#### 2.2.3. Evaluation of Selected Compounds against MMP-12, hCAII, and SaPC

5-(Heteroarylmethylene)hydantoins were evaluated as inhibitors for MMP-12 at fixed concentrations of 200 and 25 μM (Table 1). At 200 μM of **20**–**24**, the activity of MMP-12 was ≤50% the activity of positive control; for compounds **21**, **24**, and **25** at 25 μM, the activity of MMP12 was 69–74% while the activity for the others was >80%. Thus, all compounds were more potent as inhibitors for GSK-3β than for MMP-12. A select subset of the 5-(heteroarylmethylene)hydantoins were evaluated as inhibitors of human carbonic anhydrase II (*h*CAII) at 200 μM (Table 1). Only **25** and **29** exhibited any inhibition (57% and 63% activity, respectively) while the others exhibited little or no inhibitory activity. Finally, this same subset of 5-(heteroarylmethylene)hydantoins were evaluated as inhibitors of isolated *S. aureus* pyruvate carboxylase (*Sa*PC) in a fixed-time assay (Table 1) [27]. No inhibitory activity was observed up to 1000 μM.

## 3. Materials and Methods

### 3.1. Chemistry

#### 3.1.1. General Experimental Information

Melting points were measured in open capillary tubes on a MelTemp melting point apparatus and are uncorrected. NMR spectra were recorded on either a Varian Mercury+ 300 Hz or a Varian UnityInova 400 MHz instrument. d_6_-DMSO was purchased from Cambridge Isotope Laboratories. ^1^H NMR were calibrated to 2.49 ppm for d_5_-DMSO present in the d_6_-DMSO solvent, and ^13^C NMR spectra were calibrated from the central peak at 39.7 ppm for d_6_-DMSO. Coupling constants are reported in Hz. Scanned NMR spectra of compounds **15**, **17**–**29** are contained in the Supplementary Material. Elemental analyses were obtained from Midwest Microlabs, Ltd. (Indianapolis, IN, USA), and high-resolution mass spectra were obtained from the COSMIC lab at Old Dominion University. Pyridinecarboxaldehydes were purchased from Acros, Ambeed, or Ark Pharm; hydantoin was purchased from Acros. 

#### 3.1.2. General Procedure for the Preparation of 5-(Heteroarylmethylene)hydantoins

Hydantoin (10 mmol) was dissolved in H_2_O (20 mL) at 70 °C with stirring. Ethanolamine (1.0 mL) was added to the mixture, which was then heated to reflux. An equimolar quantity of the appropriate aldehyde solution (5 mmol in 5 mL ethanol) was added dropwise. The reaction mixture was heated at reflux for 5 h. The mixture was cooled to room temperature, during which time the product precipitated. The precipitate was collected via vacuum filtration, washed several times with H_2_O, and dried in vacuo. 

(*Z*)-5-[(2′-Pyridinyl)methylene]-2,4-imidazolidinedione (**17**). The reaction of pyridine-2-carboxaldehyde (1.064 g, 9.933 mmol) with hydantoin (1.005 g, 10.05 mmol) was carried out following the general procedure to give **17** as a cream-colored solid (727 mg, 38%); mp 231–233 °C (Lit. mp 233.5–235.5 °C [25]); ^1^H NMR (d_6_-DMSO, 400 MHz) δ 8.64 (d, J = 4.8 Hz, H6′), 7.80 (dt, *J* = 1.6, 7.6 Hz, H4′), 7.57 (d, *J* = 8.0 Hz, H3′), 7.27 (ddd, *J* = 0.8, 4.8, 7.6 Hz, H5′), 6.47 (s, C=CH); ^13^C NMR (d_6_-DMSO, 75 MHz) δ 165.3 (C4), 154.8, 153.8, 149.5, 137.2, 131.8, 125.6, 122.4, 105.1 (C6) ppm. The ^1^H NMR spectral data are consistent with the values in the literature [25].

(*Z*)-5-[(2-Quinolinyl)methylene]-2,4-imidazolidinedione (**18**). The reaction of quinoline-2-carboxaldehyde (0.835 g, 5.31 mmol) with hydantoin (0.539 g, 5.39 mmol) was carried out following the general procedure to give **18** as a tan solid (872 mg, 69%); mp > 255 °C (lit. mp 266–268 °C [28]); ^1^H NMR (d_6_-DMSO, 400 MHz) δ 11.43 (s, 1H, NH), 10.73 (s, 1H, NH), 8.43 (d, *J* = 8.4 Hz, 1H), 8.37 (d, *J* = 8.8 Hz, 1H), 7.94 (d, *J* = 8.0 Hz, 1H), 7.77 (t, *J* = 8.0 Hz, 1H), 7.73 (d, *J* = 8.4 Hz, 1H), 7.59 (t, *J* = 7.2 Hz, 1H), 6.44 (s, C=CH).

(*Z*)-5-[(3′-Pyridinyl)methylene]-2,4-imidazolidinedione (**19**). The reaction of pyridine-3-carboxaldehyde (0.539 g, 5.03 mmol) with hydantoin (0.493 g, 4.93 mmol) was carried out following the general procedure to give **19** as a pale yellow solid (541 mg, 58%); mp > 255 °C; ^1^H NMR (d_6_-DMSO, 400 MHz) δ 8.77 (d, *J* = 2.0 Hz, H2′), 8.48 (d, *J* = 4.8 Hz, H6′), 8.04 (d, *J* = 8.4 Hz, H4′), 7.42 (dd, *J* = 4.8, 8.0 Hz, H5′), 6.42 (s, C=CH); ^13^C NMR (d_6_-DMSO, 100 MHz) δ 165.4 (C4), 155.8, 150.4, 148.7, 135.7, 129.7, 129.2, 123.7, 104.5 (C6) ppm. The ^1^H NMR spectral data are consistent with the values in the literature [25].

(*Z*)-5-[(6′-Methoxy-2-pyridinyl)methylene]-2,4-imidazolidinedione (**15**). The reaction of 6-methoxypyridine-2-carboxaldehyde (1.388 g, 10.12 mmol) with hydantoin (1.007 g, 10.07 mmol) was carried out following the general procedure to give **15** as a pale yellow solid (1.278 g, 58%); mp 249–251 °C; ^1^H NMR (d_6_-DMSO, 400 MHz) δ 10.00–9.00 (br s, 1H, NH), 7.69 (t, *J* = 8.0 Hz, H4′), 7.17 (d, *J* = 8.0 Hz, H3′), 6.72 (d, *J* = 8.0 Hz, H5′), 6.39 (s, C=CH), 3.92 (s, OCH_3_); ^13^C NMR (d_6_-DMSO, 100 MHz) δ 165.1 (C4), 163.2, 154.4, 151.2, 139.9, 131.1, 119.3, 110.2, 105.5 (C6), 53.3 (OCH_3_) ppm. Anal. calcd. For C_10_H_9_N_3_O_3_: C: 54.79, H: 4.14, N: 19.17, Found: C: 54.23, H: 4.00, N: 19.00.

(*Z*)-5-[(3′-Methyl-2-pyridinyl)methylene]-2,4-imidazolidinedione (**20**). The reaction of 3-methylpyridine-2-carboxaldehyde (0.999 g, 8.24 mmol) with hydantoin (0.827 g, 8.27 mmol) was carried out following the general procedure to give **20** as a chartreuse yellow solid (757 mg, 45%); mp 245–248 °C; ^1^H NMR (d_6_-DMSO, 300 MHz) δ 10.60–10.40 (br s, 1H, NH), 8.48 (d, *J* = 4.7 Hz, H6′), 7.63 (d, *J* = 7.6 Hz, H4′), 7.19 (dd, *J* = 4.7, 7.6 Hz, H5′), 6.46 (s, C=CH), 2.37 (s, CH_3_); ^13^C NMR (d_6_-DMSO, 75 MHz) δ 165.4 (C4), 154.9, 152.1, 146.9, 138.3, 132.9, 132.1, 122.3, 101.4 (C6), 18.1 (CH_3_) ppm. HRMS *m*/*z* 226.0586 (calcd for C_10_H_9_N_3_O_2_+Na^+^ 226.0587).

(*Z*)-5-[(4′-Methyl-2-pyridinyl)methylene]-2,4-imidazolidinedione-HCl (**21**). The reaction 4-methylpyridine-2-carboxaldehyde (0.971 g, 8.02 mmol) with hydantoin (0.803 g, 8.03 mmol) was carried out following the general procedure. The reaction mixture was treated with conc. HCl (15 drops) to induce the precipitation of the hydrochloride salt, which gave **21** as a cream-colored solid (677 mg, 42%); mp 225–230 °C; ^1^H NMR (d_6_-DMSO, 400 MHz) δ 8.48 (d, *J* = 4.8 Hz, H6′), 7.42 (s, H3′), 7.12 (d, *J* = 4.8 Hz, H5′), 6.41 (s, C=CH), 2.31 (s, CH_3_); ^13^C NMR (d_6_-DMSO, 100 MHz) δ 165.2 (C4), 154.7, 153.5, 149.2, 147.9, 131.8, 126.2, 123.2, 105.1 (C6), 20.5 (CH_3_) ppm. HRMS *m*/*z* 226.0585 (calcd for C_10_H_9_N_3_O_2_+Na^+^ 226.0587).

(*Z*)-5-[(5′-Methyl-2-pyridinyl)methylene]-2,4-imidazolidinedione (**22**). The reaction of 5-methylpicolinaldehyde (1.24 g, 10.24 mmol) with hydantoin (1.007 g, 10.07 mmol) was carried out following the general procedure to give **22** as a light yellow solid (987 mg, 48%); mp >250 °C; ^1^H NMR (d_6_-DMSO, 400 MHz) δ 11.34–11.26 (br s, 1H, NH), 10.32–10.26 (br s, 1H, NH), 8.50 (d, *J* = 1.2 Hz, H6′), 7.66 (dd, *J* = 1.2, 7.6 Hz, H4′), 7.51 (d, *J* = 7.6 Hz, H3′), 6.46 (s, C=CH), 2.32 (s, CH_3_); ^13^C NMR (d_6_-DMSO, 100 MHz) δ 165.2 (C4), 154.7, 151.0, 149.8, 137.4, 131.9, 131.0, 125.1, 105.2 (C6), 18.0 (CH_3_) ppm. HRMS *m*/*z* 226.0585 (calcd for C_10_H_9_N_3_O_2_+Na^+^ 226.0587).

(*Z*)-5-[(6′-Methyl-2-pyridinyl)methylene]-2,4-imidazolidinedione-HCl (**23**). The reaction of 6-methylpyridine-2-carboxaldehyde (2.395 g, 19.77 mmol) with hydantoin (1.989 g, 19.89 mmol) was carried out following the general procedure. The aqueous reaction mixture was treated with conc. HCl (8 drops) to induce the precipitation of the hydrochloride salt, which gave **23** as a pale yellow solid (1.731 g, 45%); mp 229–230 °C; ^1^H NMR (d_6_-DMSO, 300 MHz) δ 7.67 (t, *J* = 7.5 Hz, H4′), 7.36 (d, *J* = 7.5 Hz, H3′), 7.11 (d, *J* = 7.5 Hz, H5′) 6.42 (s, C=CH), 2.54 (s, CH_3_); ^13^C NMR (d_6_-DMSO, 100 MHz) δ 165.2 (C4), 158.3, 154.6, 153.1, 137.3, 131.3, 123.0, 122.1, 105.3 (C6), 24.1 (CH_3_) ppm. HRMS *m*/*z* 226.0586 (calcd for C_10_H_9_N_3_O_2_+Na^+^ 226.0587).

(*Z*)-5-[(3′-Chloro-2-pyridinyl)methylene]-2,4-imidazolidinedione-HCl (**24**). The reaction of 2-chloropyridine-2-carboxaldehyde (990 mg, 6.99 mmol) with hydantoin (699 mg, 6.99 mmol) was carried out following the general procedure. The aqueous reaction mixture was treated with conc. HCl (8 drops) to induce the precipitation of the hydrochloride salt, which gave **24** as a tan solid (1.075 g, 59%); mp 235–240 °C; ^1^H NMR (d_6_-DMSO, 400 MHz) δ 11.30–10.30 (br s, 1H, NH), 8.58 (d, *J* = 4.8 Hz, H6′), 7.96 (d, *J* = 8.0 Hz, H4′), 7.32 (dd, *J* = 4.8, 8.2 Hz, H5′), 6.61 (s, C=CH); ^13^C NMR (d_6_-DMSO, 100 MHz) δ 165.3 (C4), 155.2, 150.4, 147.9, 137.6, 133.8, 129.9, 123.5, 98.8 (C6) ppm; Anal. calcd. For C_9_H_6_ClN_3_O_2_-0.6HCl: C: 44.03, H: 2.71, N: 17.12, Found: C: 44.22, H: 2.63, N: 16.97.

(*Z*)-5-[(4′-Chloro-2-pyridinyl)methylene]-2,4-imidazolidinedione-HCl (**25**). The reaction of 4-chloropyridine-2-carboxaldehyde (1.02 g, 7.21 mmol) with hydantoin (0.720 g, 7.20 mmol) was carried out following the general procedure. The aqueous solution was treated with conc. HCl (6 drops) to induce the precipitation of the hydrochloride salt (**25**) which was obtained as a bright yellow solid (580 mg, 31%); mp 210–215 °C (dec); ^1^H NMR (d_6_-DMSO, 400 MHz) δ 11.10-10.04 (br s, 1H, NH), 8.57 (d, *J* = 5.4 Hz, H6′), 7.76 (d, *J* = 2.0 Hz, H3′), 7.39 (dd, *J* = 2.0, 5.4 Hz, H5′), 6.46 (s, C=CH); ^13^C NMR (d_6_-DMSO, 100 MHz) δ 165.1 (C4), 155.5, 154.9, 150.7, 143.5, 132.9, 124.8, 122.0, 103.6 (C6) ppm. HRMS *m*/*z* 246.0039 (calcd for formula C_9_H_6_ClN_3_O_2_+Na^+^ 246.0041).

(*Z*)-5-[(5′-Bromo-2-pyridinyl)methylene]-2,4-imidazolidinedione (**26**). The reaction of 5-bromo-2-pyridinecarboxaldehyde (1.918 g, 10.31 mmol) with hydantoin (1.017 g, 10.17 mmol) was carried out following the general procedure to give **26** as a tan-colored solid (1.504 g, 55%); mp > 255 °C; (d_6_-DMSO, 400 MHz) δ 8.70 (d, *J* = 2.4 Hz, H6′), 8.08 (dd, *J* = 2.4, 8.6 Hz, H4′), 7.58 (d, *J* = 8.6 Hz, H3′), 6.48 (s, C=CH); ^13^C NMR d_6_-DMSO, 100 MHz) δ 165.7 (C4), 155.4, 152.9, 150.4, 140.0, 132.7, 127.2, 118.7, 104.2 (C6) ppm; Anal. calcd. For C_9_H_6_BrN_3_O_2_: C: 40.32, H: 2.26, N: 15.68, Found: C: 40.17, H: 2.25, N: 15.41.

(*Z*)-5-[(6′-Bromo-2-pyridinyl)methylene]-2,4-imidazolidinedione (**27**). The reaction 6-bromo-2-pyridinecarboxaldehyde (1.855 g, 9.97 mmol) with hydantoin (1.026 g, 10.26 mmol) was carried out following the general procedure to give **27** as a pale yellow solid (1.357 g, 48%); mp > 250 °C; ^1^H NMR (d_6_-DMSO, 400 MHz) δ 7.73 (t, *J* = 7.6 Hz, H4′), 7.59 (d, *J* = 7.6 Hz, H5′), 7.50 (d, *J* = 7.6 Hz, H3′), 6.39 (s, C=CH); ^13^C NMR d_6_-DMSO, 100 MHz) δ 165.0 (C4), 154.9, 154.7, 140.9, 140.2, 132.3, 126.5, 124.7, 103.5 (C6) ppm; HRMS *m*/*z* 289.9534 (calcd for C_9_H_6_NrN_3_O_2_+Na^+^ 289.9536). Anal. calcd. For C_9_H_6_BrN_3_O_2_-0.6H_2_O: C: 38.76, H: 2.60, N: 15.06, Found: C: 38.81, H: 2.38, N: 14.71.

(*Z*)-5-[(6′-Quinolinyl)methylene]-2,4-imidazolidinedione (**28**). The reaction of 6-quinolinecarboxaldehyde (1.58 g, 10.0 mmol) with hydantoin (1.08 g, 10.8 mmol) was carried out following the general procedure to give **28** as a peach-colored solid (0.92 g, 37%); mp > 250 °C; ^1^H NMR (d_6_-DMSO, 400 MHz) δ 11.50-10.00 (br s, 1H, NH), 8.88 (d, *J* = 4.0 Hz, H2′), 8.32 (d, *J* = 8.4 Hz, H4′), 8.26 (s, H5′), 7.99 and 7.93 (AB, *J_AB_* = 8.8 Hz, H7′ and H8′), 7.55 (dd, J = 4.0, 8.4 Hz, H3′), 6.57 (s, C=CH); ^13^C NMR (d_6_-DMSO, 100 MHz) δ 165.6 (C4), 155.9, 151.1, 147.1, 136.3, 131.2, 130.9, 129.2, 129.0, 128.4, 128.0, 122.1, 107.3 (C6) ppm. HRMS *m*/*z* 240.0766 (calcd for formula C_13_H_9_N_3_O_2_+H^+^ 240.0768).

(*Z*)-5-[(3-Benzo[b]thiophenyl)methylene]-2,4-imidazolidinedione (**29**). The reaction of benzo[b]thiophene-3-carboxaldehyde (1.62 g, 9.99 mmol) with hydantoin (0.995 g, 9.95 mmol) was carried out following the general procedure to give **29** as a yellow solid (1.465 g, 60%); mp > 250 °C; ^1^H NMR (d_6_-DMSO, 400 MHz) δ 11.20–10.80 (br s, 1H, NH), 8.24 (s, 1H), 8.04 (d, *J* = 8.0 Hz, 1H), 7.99 (d, *J* = 7.6 Hz, 1H), 7.51–7.41(m, 2H), 6.71 (s, 1H, C=CH); ^13^C NMR (d_6_-DMSO, 100 MHz) δ 165.4, 155.7, 138.7, 138.0, 128.9, 127.9, 127.0, 125.2, 125.0, 123.1, 121.6, 98.9 (C6) ppm. HRMS *m*/*z* 267.0198 (calcd for formula C_12_H_8_N_2_O_2_S+Na^+^ 267.0199).

### 3.2. Biological Evaluation

#### 3.2.1. Inhibition of Glycogen Synthase Kinase-3β

In vitro inhibition assays of glycogen synthase kinase-3β were performed by Reaction Biology using their HotSpot^TM^ assay as previously described [31]. The following description is similar to that in the reference. Briefly, the GSK-3β kinase and each compound along with required cofactors were prepared in reaction buffer (20 mM Hepes pH 7.5, 10 mM MgCl_2_, 1 mM EGTA, 0.01% Brij35, 0.02 mg/mL BSA, 0.1 mM Na_3_VO_4_, 2 mM DTT, 1% DMSO). Compounds were delivered into the reaction. After 20 min, a mixture of ATP and ^33^P ATP was prepared to a final concentration of 10 μM. After reaction for 120 min at 25 °C, the mixture was spotted onto P81 ion exchange filter paper. Unbound phosphate was removed by means of extensive washing with 0.75% phosphoric acid. Background derived from control reactions with the inactive enzyme was subtracted and the kinase activity expressed as percent remaining kinase activity compared to vehicle. Percent inhibition at a single concentration was conducted in duplicate at a 25 μM inhibitor; values are reported as the average. IC_50_ determinations were conducted in triplicate; and curve fits were obtained using Prism. Values are reported as the average with calculated standard deviation. Staurosporine was tested in a 10-dose IC_50_ 4-fold serial dilution as a control; this gave an IC_50_ value of 5.12 nM.

#### 3.2.2. Inhibition of Matrix Metalloproteinase-12

Assays for human matrix metalloproteinase-12 activity were performed in a 96-well plate format for a total reaction volume of 100 μL, in an assay modified from Day and Cohen [32]. All assay reagents were prepared and maintained at 22 °C until warmed to 37 °C. The omniMMP fluorogenic substrate and MMP-12 enzyme were prepared in assay buffer (50 mM HEPES, 10 mM CaCl_2_, 0.05% Brij-35, pH of 7.52), and small molecule effectors were prepared as a solution in 50% DMSO, 50% assay buffer. The final concentration of enzyme in the 100 μL enzymatic reaction was 0.0083 U/μL. A known inhibitor, *N*-[(4’-bromo [1,1’-biphenyl]-4-yl)sulfonyl]-L-valine (PD166793), was included where noted at a final concentration of 100 μM. OmniMMP fluorogenic substrate was added to the 100 μL enzymatic reaction at a final concentration of 4 μM. For all experiments, 30 μL of enzyme stock solution was added to each well in a 96-well, flat-bottom, black polystyrene microplate (SantaCruz Biotechnology, Dallas, TX, USA), followed by the addition of 10 μL of the effectors (50% assay buffer, 50% DMSO for the uninhibited reaction, PD166793 for the inhibition). The 96-well plate was then incubated in a plate reader at 37 °C for 30 min; concurrently, the substrate solution was also warmed to 37 °C for 30 min. The enzymatic reaction was initiated by adding 60 μL of the omniMMP fluorogenic substrate solution. The change in fluorescence was then monitored for 30 min with excitation and emission wavelengths at 320 and 400 nm, respectively, every 46 s for a total of 40 measurements.

#### 3.2.3. Inhibition of Human Carbonic Anhydrase II

Assays for human carbonic anhydrase II activity were performed in a 96-well plate format for a total reaction volume of 100 μL, in an assay modified from Day and Cohen [32]. All assay reagents and solutions were prepared separately and maintained at 22 °C until warmed to 30 °C. All substrates, effectors, and enzyme were freshly dissolved and diluted in assay buffer containing 50 mM Tris (pH 8.0). The final concentration of the human carbonic anhydrase II enzyme was 200 nM. Where noted, acetazolamide was included at a final concentration of 10 μM. The substrate, *p*-nitrophenyl acetate, was added at a final concentration of 504 μM. For all experiments, 20 μL of enzyme stock solution was added to each well in a 96-well, flat-bottom, polystyrene microplate (Santa Cruz Biotechnology, Dallas, TX, USA), followed by the addition of 10 μL of the effectors (assay buffer for the uninhibited reaction, acetazolamide for the inhibition). The 96-well plate was then incubated in a plate reader at 30 °C for 10 min; concurrently, the substrate solution was also warmed to 30 °C for 10 min. The enzymatic reaction was initiated by adding 70 μL of the substrate solution (p-nitrophenyl acetate). The absorbance values were then measured at 405 nm every 30 s over a period of 20 min for a total of 40 measurements.

#### 3.2.4. Inhibition of *S. aureus* Pyruvate Carboxylase (SaPC)

Malate dehydrogenase-coupled enzyme assays were performed at 22 °C in a 96-well plate format for a total reaction volume of 200 μL. Assay conditions consisted of 100 mM Tris (pH 7.8), 7 mM MgCl_2_, 150 mM KCl, and 0.5% Triton x-100. First, 20 μL of compound was added to obtain the desired final concentration; 20 μL of malate dehydrogenase (MDH) was added such that the final concentration in the assay was 20 U/mL; and lastly, 140 μL of substrates was added to initiate the reaction (HCO_3−_, ATP, and NADH to a final assay concentration of 15 mM, 2.5 mM, and 0.25 mM, respectively). To account for the possible compound inhibition of MDH, the procedure was modified such that 20 μL oxaloacetate was added to a final concentration of 30 mM, in place of PC. Reagents were dispensed manually by a hand-held, multi-channel micropipette, and absorbance measurements were recorded at 340 nM with a Molecular Devices SpectraMax i3x Multi-mode plate reader.

## 4. Conclusions

Several 5-(heteroarylmethylene)hydantoins were found to be single-digit micromolar inhibitors of GSK-3β, with **25** and **27** as the most potent ones. Computational docking predicted that these bind in the ATP binding domain, with hydrogen bonding between the hydantoin functionality and the backbone C=O and N–H of a valine 135 in this domain. The GSK-3β inhibitory activities of the compounds reported in this manuscript are in the same range as previously reported for substituted 5-phenylmethylenehydantoins (**1**–**8**) [23], and they are considerably less potent than the highly potent inhibitors stauroporine (IC_50_ = 15 nM), indirubin-3-monoxime (IC_50_ = 22 nM), and alsterpaullone (IC_50_ = 4 nM). Developing more potent inhibitors based on this scaffold will likely require the introduction of additional functionality which can take advantage of hydrogen bonding and/or lipophilic functionality present within the binding pocket. Certain of these compounds exhibited an inhibition of MMP-12 at 200 μM, and further inhibitor design may be needed to improve the selectivity for GSK-3β over MMP-12; there was essentially no inhibitory activity observed for *h*CAII or *Sa*PC.

## Figures and Tables

**Table 1:** Inhibitory activity of 5-(heteroarylmethylene)hydantoins.

Compd	GSK-3β % Activity @ 25 μM	GSK-3β IC_50_	GSK-3β Docking Score ^1^	MMP-12 % Activity @ 200 μM/25 μM	*h*CAII % Activity @ 200 μM	*Sa*PC IC_50_
**17**	55.4	ND	–7.1	ND	ND	ND
**18**	32.9	ND	–8.7	57.6/94.1	ND	>1000 μM
**19**	71.8	ND	–7.1	50.6/84.2	88.9 ± 12.3	>1000 μM
**15**	17.3	4.06 ± 0.30 μM	–7.4	74.0/87.5	82.1 ± 11.0	>1000 μM
**20**	31.6	ND	–7.6	39.6/108	ND	ND
**21**	42.0	ND	–7.6	28.5/71.6	94.8 ± 12.8	>1000 μM
**22**	47.0	ND	–7.3	40.7/101	87.4 ± 11.8	>1000 μM
**23**	28.4	8.34 ± 0.27 μM	–7.4	46.5/86.4	88.9 ± 12.3	>1000 μM
**24**	44.6	ND	–7.2	39.2/69.1	91.8 ± 14.7	>1000 μM
**25**	15.4	2.14 ± 0.18 μM	–7.5	50.0/73.9	57.0 ± 16.4	>1000 μM
**26**	35.3	ND	–6.7	72.9/98.6	ND	ND
**27**	17.0	3.39 ± 0.16 μM	–7.5	88.7/85.8	86.7 ± 12.1	>1000 μM
**28**	22.5	7.82 ± 0.12 μM	–8.8	61.6/81.8	ND	ND
**29**	27.9	ND	–7.8	88.0/98.1	62.7 ± 9.8	>1000 μM

^1^ Docking scores were calculated using Mcule online one-click with the Vina docking algorithm (https://mcule.com, accessed on 8 February 2024). ND signifies “not determined”.

## Data Availability

The data presented in this study are available on request from the corresponding author due to legal reasons.

## Supplementary Materials

The following supporting information can be downloaded at: https://www.mdpi.com/article/10.3390/ph17050570/s1, ^1^H and/or ^13^C NMR spectra of synthesized compounds.
