# Supplementary material for: Synthesis and Evaluation of 5-(Heteroarylmethylene)hydantoins as Glycogen Synthase Kinase-3β Inhibitors
Source: Pharmaceuticals (Basel). 2024 Apr 29;17(5):570. doi: 10.3390/ph17050570 (PMC11123921; doi:10.3390/ph17050570)
Supplement: Supplementary file 1 [file pharmaceuticals-17-00570-s001.zip › pharmaceuticals-2939237-supplementary.pdf]

Supplementary Material for

Synthesis and Evaluation of 5-(Heteroarylmethylene)hydantoins as Glycogen Synthase Kinase-3β Inhibitors

Nicholas O. Schneider, Kendra Gilreath, Daniel J. Burkett, Martin St. Maurice and William A. Donaldson

This document includes:

^{1}H NMR spectrum of **17** (d_6-DMSO) S2
^{13}C NMR spectrum of **17** (d_6-DMSO) S3
^{1}H NMR spectrum of **18** (d_6-DMSO) S4
^{1}H NMR spectrum of **19** (d_6-DMSO) S5
^{13}C NMR spectrum of **19** (d_6-DMSO) S6
^{1}H NMR spectrum of **15** (d_6-DMSO) S7
^{13}C NMR spectrum of **15** (d_6-DMSO) S8
^{1}H NMR spectrum of **20** (d_6-DMSO) S9
^{13}C NMR spectrum of **20** (d_6-DMSO) S10
^{1}H NMR spectrum of **21** (d_6-DMSO) S11
^{13}C NMR spectrum of **21** (d_6-DMSO) S12
^{1}H NMR spectrum of **22** (d_6-DMSO) S13
^{13}C NMR spectrum of **22** (d_6-DMSO) S14
^{1}H NMR spectrum of **23** (d_6-DMSO) S15
^{13}C NMR spectrum of **23** (d_6-DMSO) S16
^{1}H NMR spectrum of **24** (d_6-DMSO) S17
^{13}C NMR spectrum of **24** (d_6-DMSO) S18
^{1}H NMR spectrum of **25** (d_6-DMSO) S19
^{13}C NMR spectrum of **25** (d_6-DMSO) S20
^{1}H NMR spectrum of **26** (d_6-DMSO) S21
^{13}C NMR spectrum of **26** (d_6-DMSO) S22
^{1}H NMR spectrum of **27** (d_6-DMSO) S23
^{13}C NMR spectrum of **27** (d_6-DMSO) S24
^{1}H NMR spectrum of **28** (d_6-DMSO) S25
^{13}C NMR spectrum of **28** (d_6-DMSO) S26
^{1}H NMR spectrum of **29** (d_6-DMSO) S27
^{13}C NMR spectrum of **29** (d_6-DMSO) S28

SpinWorks 4: DJB-710-proton-DMSO-overnight-reflux

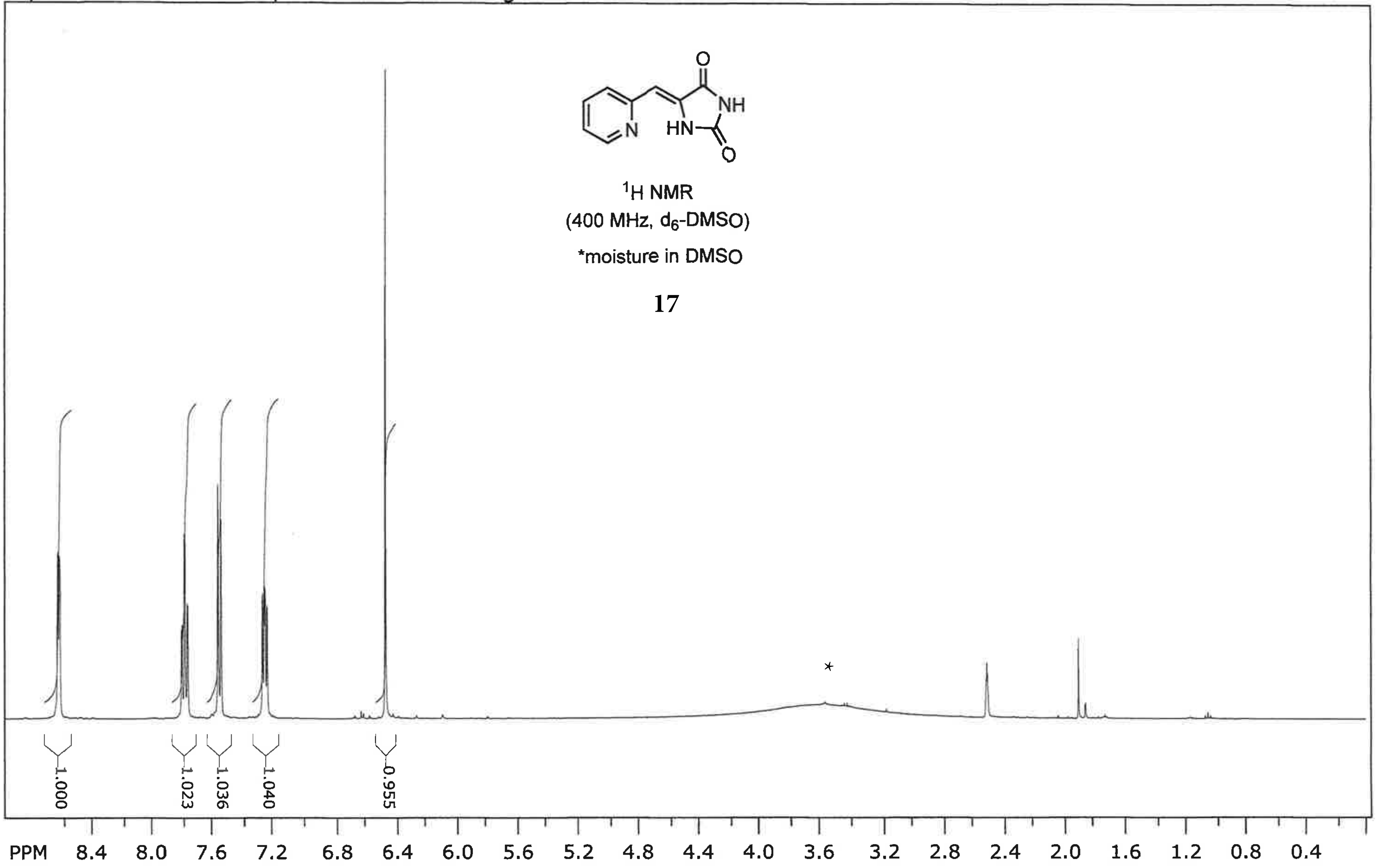

file: ...oton-DMSO-reflux-overnight.fid\fid block# 1 expt: "s2pul"
transmitter freq.: 399.732139 MHz
time domain size: 32768 points
width: 6410.26 Hz = 16.0364 ppm = 0.195626 Hz/pt
number of scans: 8

freq. of 0 ppm: 399.729732 MHz
processed size: 32768 complex points
LB: 0.500 GF: 0.0000
Hz/cm: 143.875 ppm/cm: 0.35993

SpinWorks 4: 13C OBSERVE

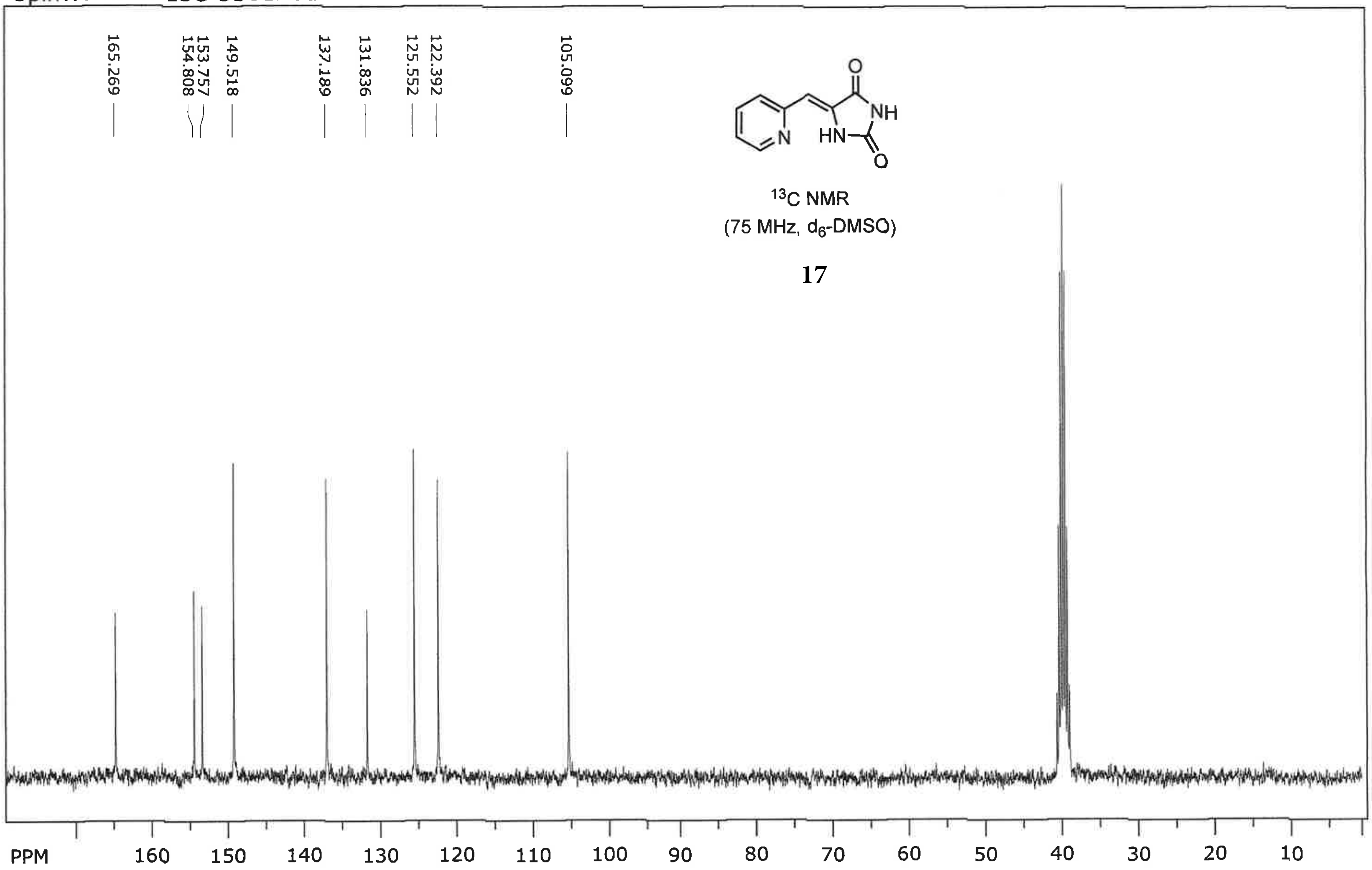

file: ...ldsonw\Desktop\DJB-234-13C.fid\fid block# 1 expt: "s2pul"
transmitter freq.: 75.476694 MHz
time domain size: 68492 points
width: 18867.92 Hz = 249.9835 ppm = 0.275476 Hz/pt
number of scans: 256

freq. of 0 ppm: 75.468435 MHz
processed size: 131072 complex points
LB: 2.500 GF: 0.0000
Hz/cm: 543.396 ppm/cm: 7.19952

SpinWorks 4: DJB-331-proton

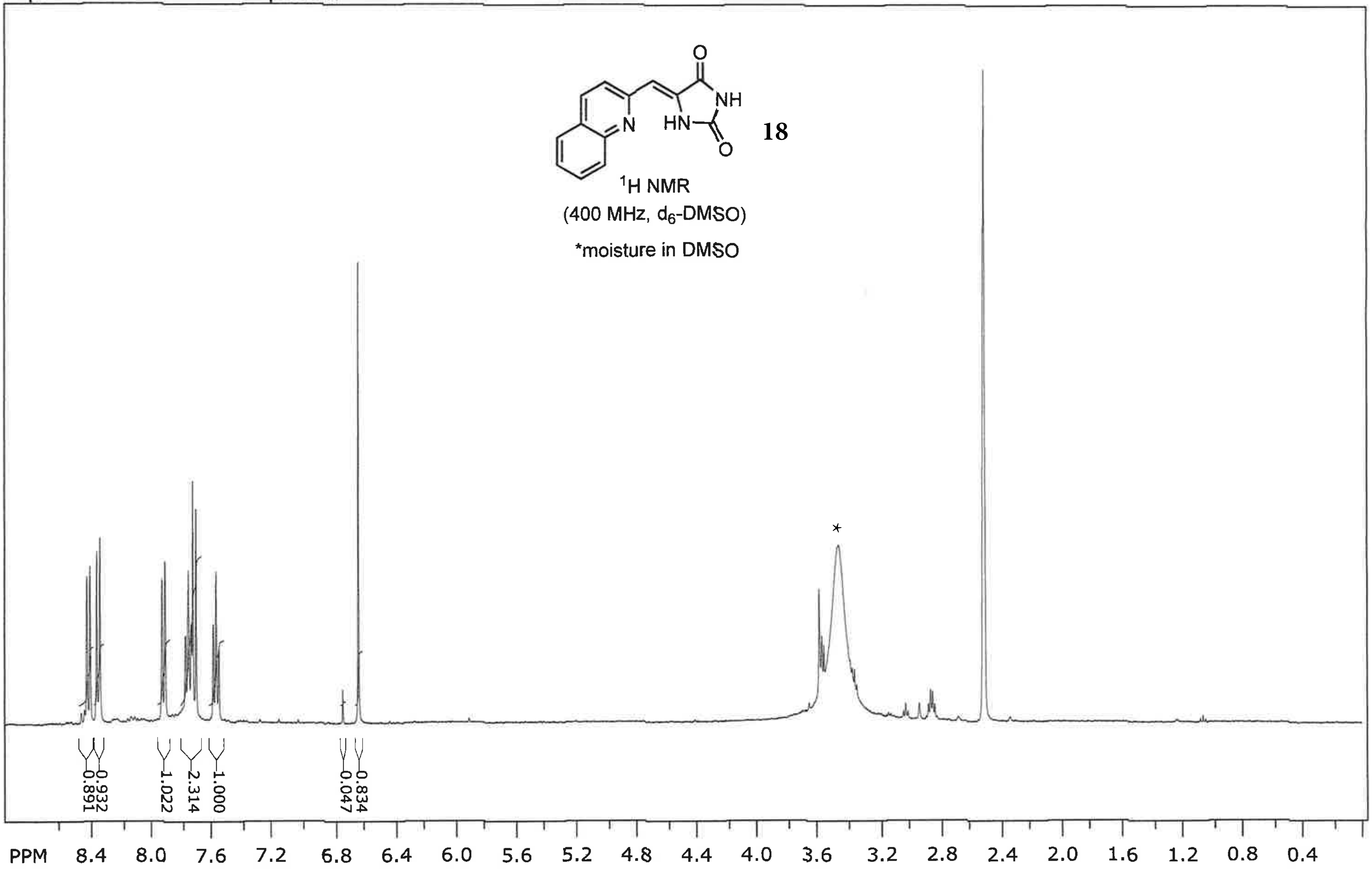

file: ...DJB spectra\DJB-334-proton.fid\fid block# 1 expt: "s2pul"
transmitter freq.: 399.736048 MHz
time domain size: 32768 points
width: 6410.26 Hz = 16.0362 ppm = 0.195626 Hz/pt
number of scans: 8

freq. of 0 ppm: 399.733634 MHz
processed size: 32768 complex points
LB: 0.500 GF: 0.0000
Hz/cm: 143.875 ppm/cm: 0.35992

SpinWorks 4:

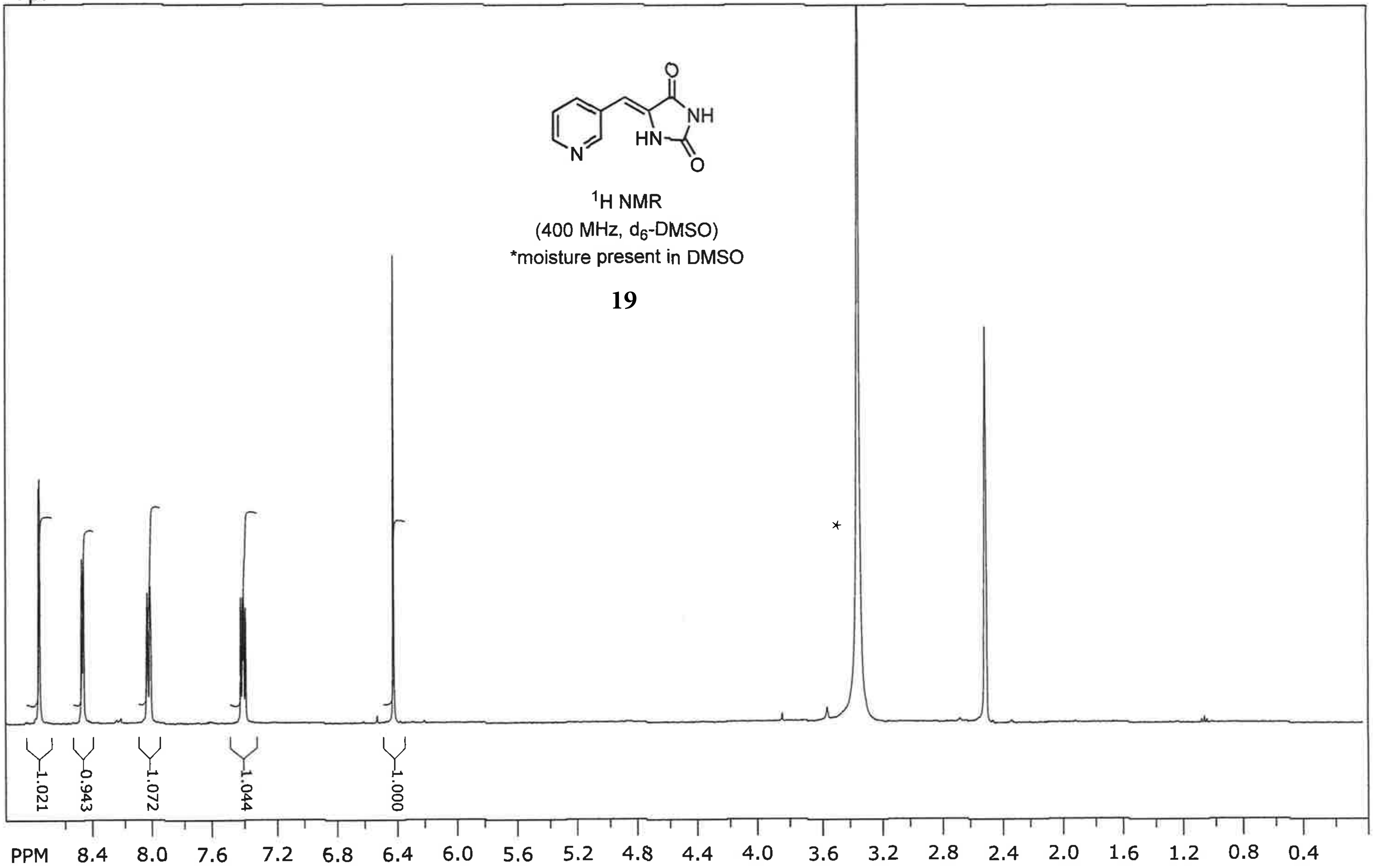

file: ...DJB spectra\DJB-235-proton.fid\fid block# 1 expt: "s2pul"
transmitter freq.: 399.738002 MHz
time domain size: 26264 points
width: 6410.26 Hz = 16.0361 ppm = 0.244070 Hz/pt
number of scans: 8

freq. of 0 ppm: 399.735598 MHz
processed size: 65536 complex points
LB: 0.500 GF: 0.0000
Hz/cm: 143.875 ppm/cm: 0.35992

SpinWorks 4: JC_SS_1

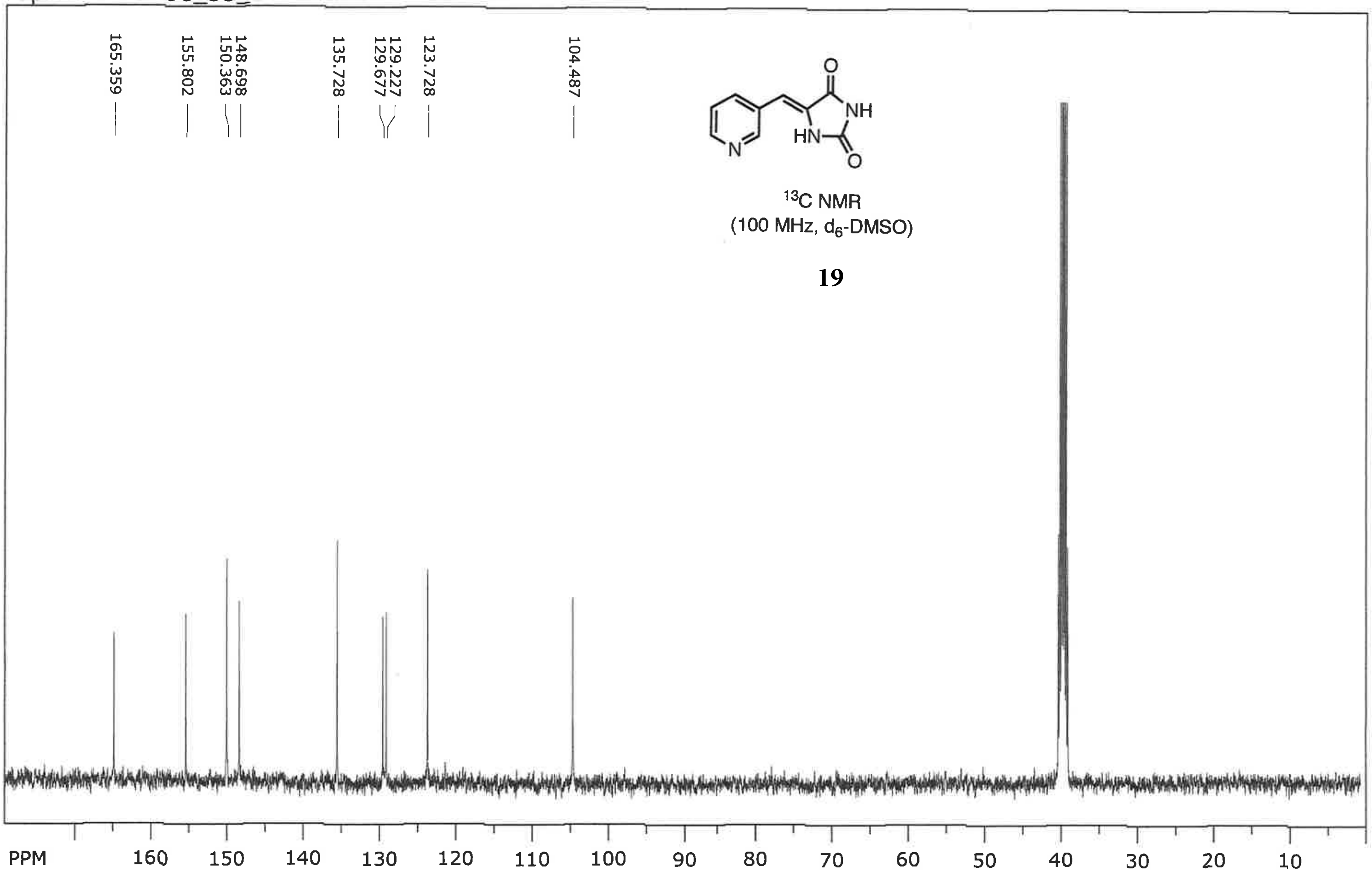

file: ...on\WD325_13C_DMSO_20240116.fid\fid block# 1 expt: "s2pul"
transmitter freq.: 100.523180 MHz
time domain size: 65536 points
width: 25000.00 Hz = 248.6989 ppm = 0.381470 Hz/pt
number of scans: 2048

freq. of 0 ppm: 100.512160 MHz
processed size: 65536 complex points
LB: 2.500 GF: 0.0000
Hz/cm: 723.333 ppm/cm: 7.19569

SpinWorks 4: 7_14_20_PentylClick

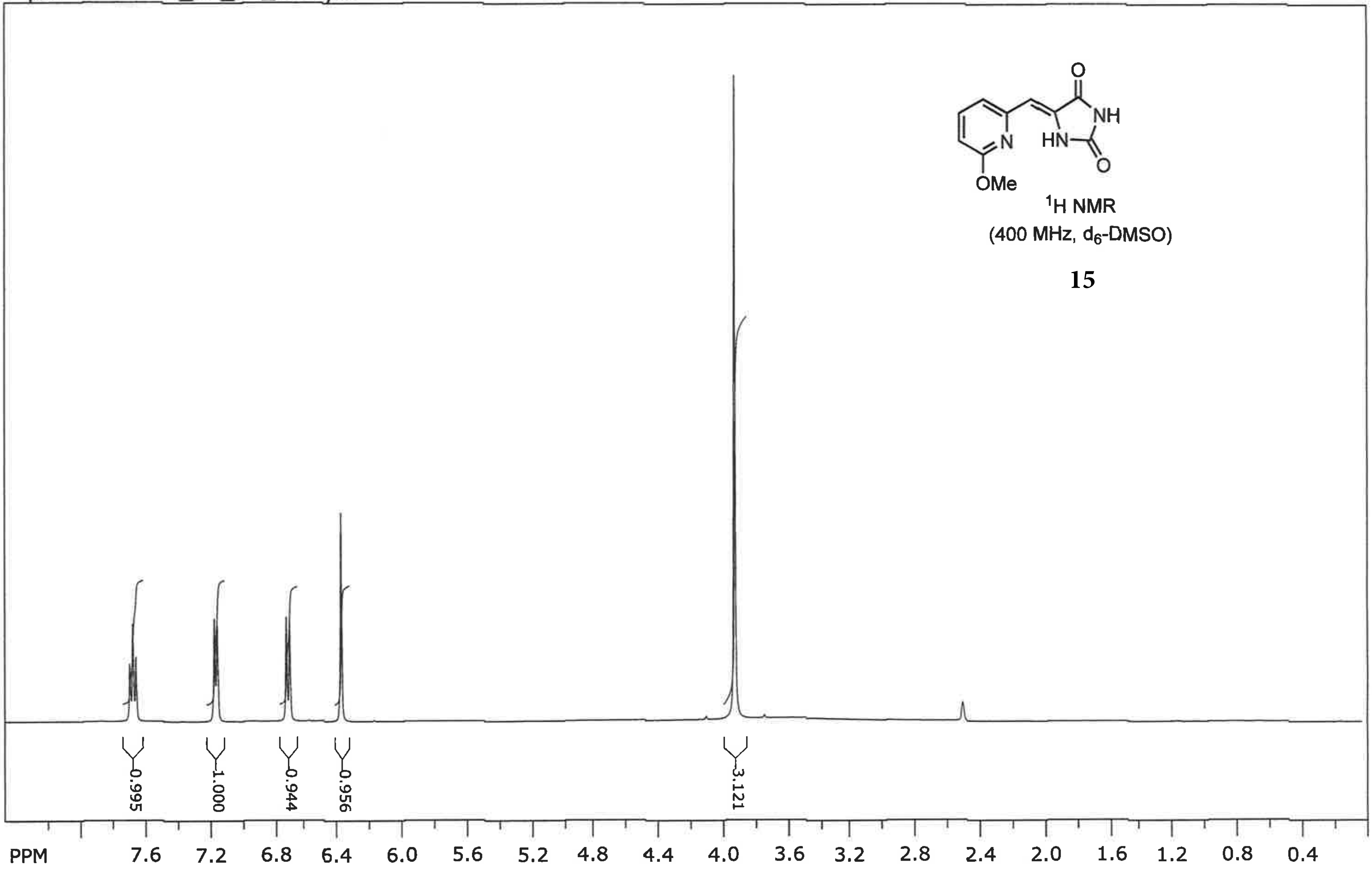

^{1}H NMR
(400 MHz, d_6-DMSO)

15

file: ...spectra\WD340-349\WD340-1H.fid\fid block# 1 expt: "s2pul"
transmitter freq.: 399.732139 MHz
time domain size: 32768 points
width: 6410.26 Hz = 16.0364 ppm = 0.195626 Hz/pt
number of scans: 8

freq. of 0 ppm: 399.729734 MHz
processed size: 32768 complex points
LB: 0.500 GF: 0.0000
Hz/cm: 135.897 ppm/cm: 0.33997

SpinWorks 4: 7_27_20_Ester_carbon

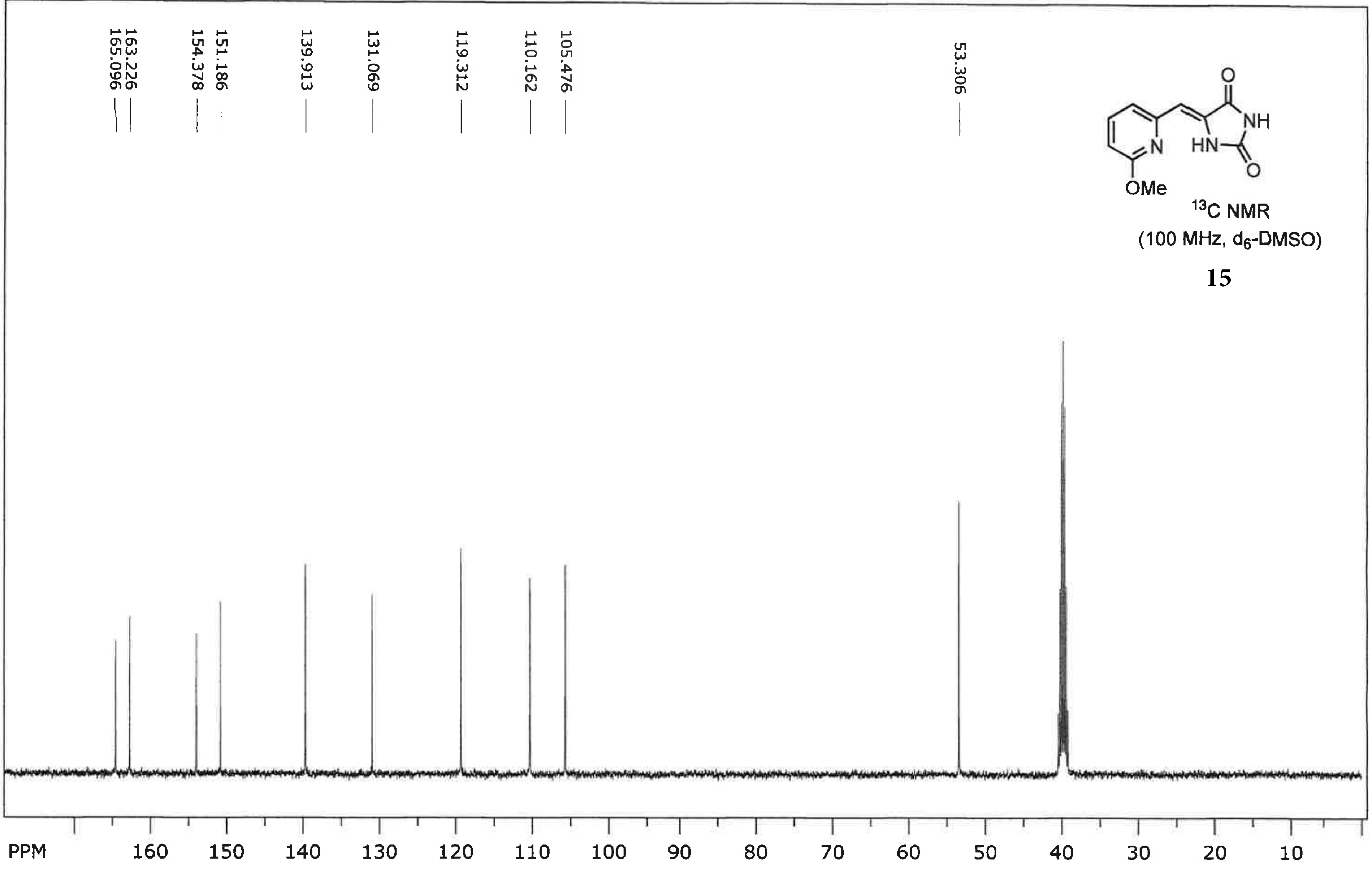

file: ...ectra\WD spectra\WD340-13C.fid\fid block# 1 expt: "s2pul"
transmitter freq.: 100.523180 MHz
time domain size: 65536 points
width: 25000.00 Hz = 248.6989 ppm = 0.381470 Hz/pt
number of scans: 256

freq. of 0 ppm: 100.512156 MHz
processed size: 65536 complex points
LB: 1.500 GF: 0.0000
Hz/cm: 723.889 ppm/cm: 7.20121

SpinWorks 4: STANDARD 1H OBSERVE

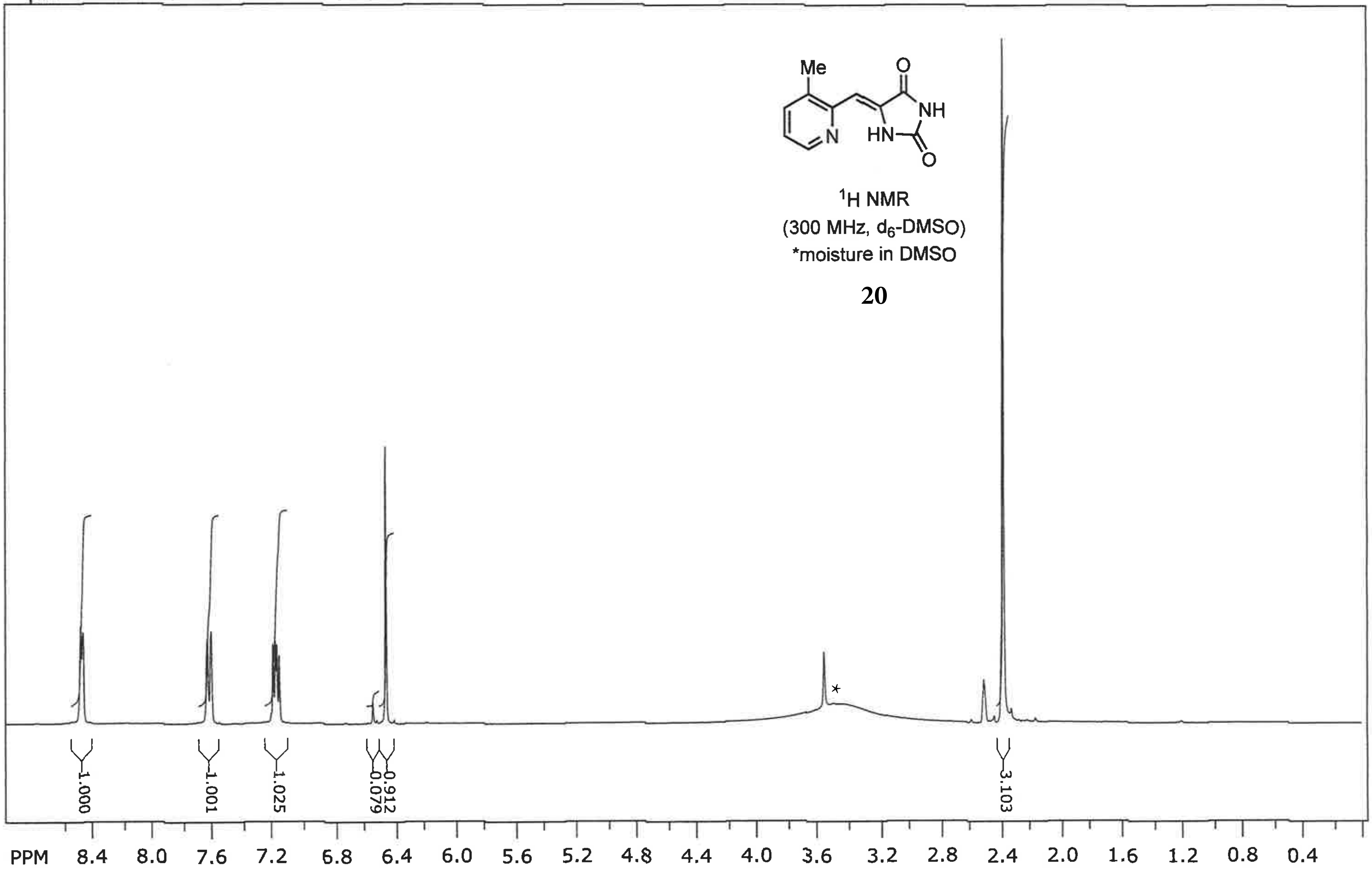

file: ...pectra\WD320-329\WD-328_1H.fid\fid block# 1 expt: "s2pul"
transmitter freq.: 300.134434 MHz
time domain size: 19192 points
width: 4803.07 Hz = 16.0031 ppm = 0.250264 Hz/pt
number of scans: 8

freq. of 0 ppm: 300.132628 MHz
processed size: 32768 complex points
LB: 0.500 GF: 0.0000
Hz/cm: 108.016 ppm/cm: 0.35989

SpinWorks 4: 13C OBSERVE

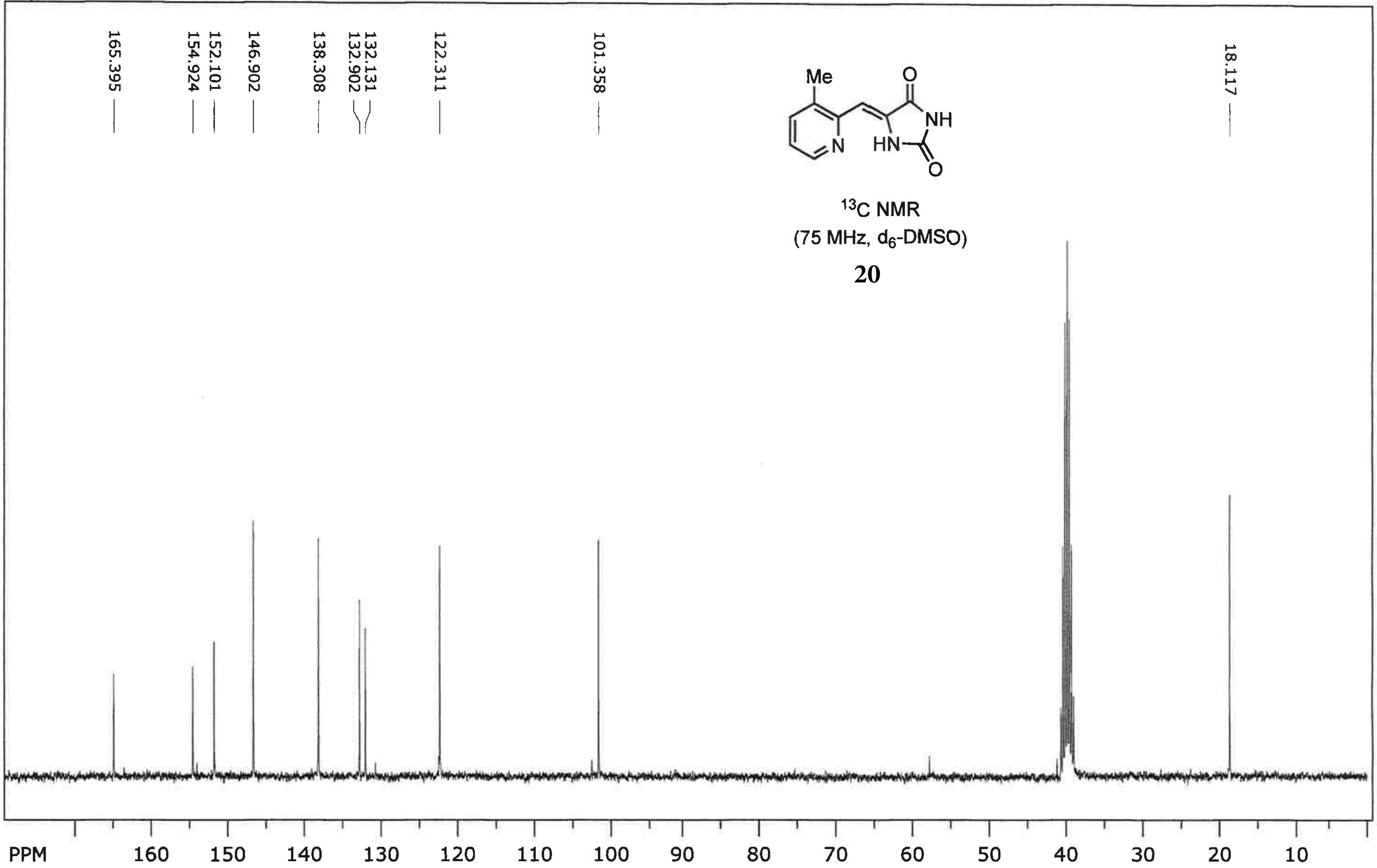

file: ...ctra\WD spectra\WD-328_13C.fid\fid block# 1 expt: "s2pul"
transmitter freq.: 75.476694 MHz
time domain size: 68492 points
width: 18867.92 Hz = 249.9835 ppm = 0.275476 Hz/pt
number of scans: 384

freq. of 0 ppm: 75.468436 MHz
processed size: 131072 complex points
LB: 1.500 GF: 0.0000
Hz/cm: 543.396 ppm/cm: 7.19952

SpinWorks 4: 7_30_20_Cyclobutanone_carbon

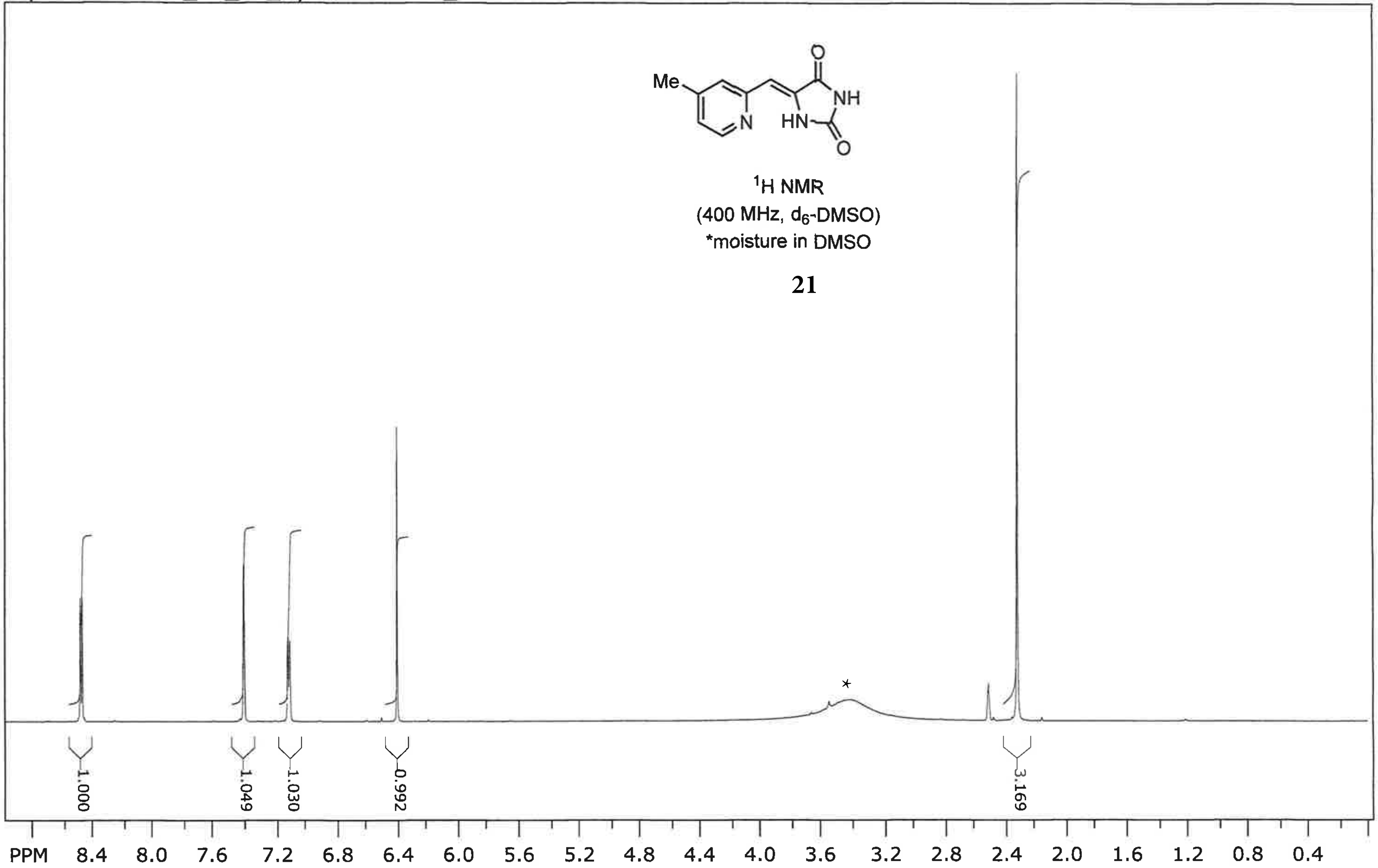

file: ...pectra\WD spectra\WD342_1H.fid\fid block# 1 expt: "s2pul"
transmitter freq.: 399.732139 MHz
time domain size: 32768 points
width: 6410.26 Hz = 16.0364 ppm = 0.195626 Hz/pt
number of scans: 8

freq. of 0 ppm: 399.729733 MHz
processed size: 32768 complex points
LB: 0.051 GF: 0.0000
Hz/cm: 144.017 ppm/cm: 0.36028

SpinWorks 4: STANDARD 1H OBSERVE - profile

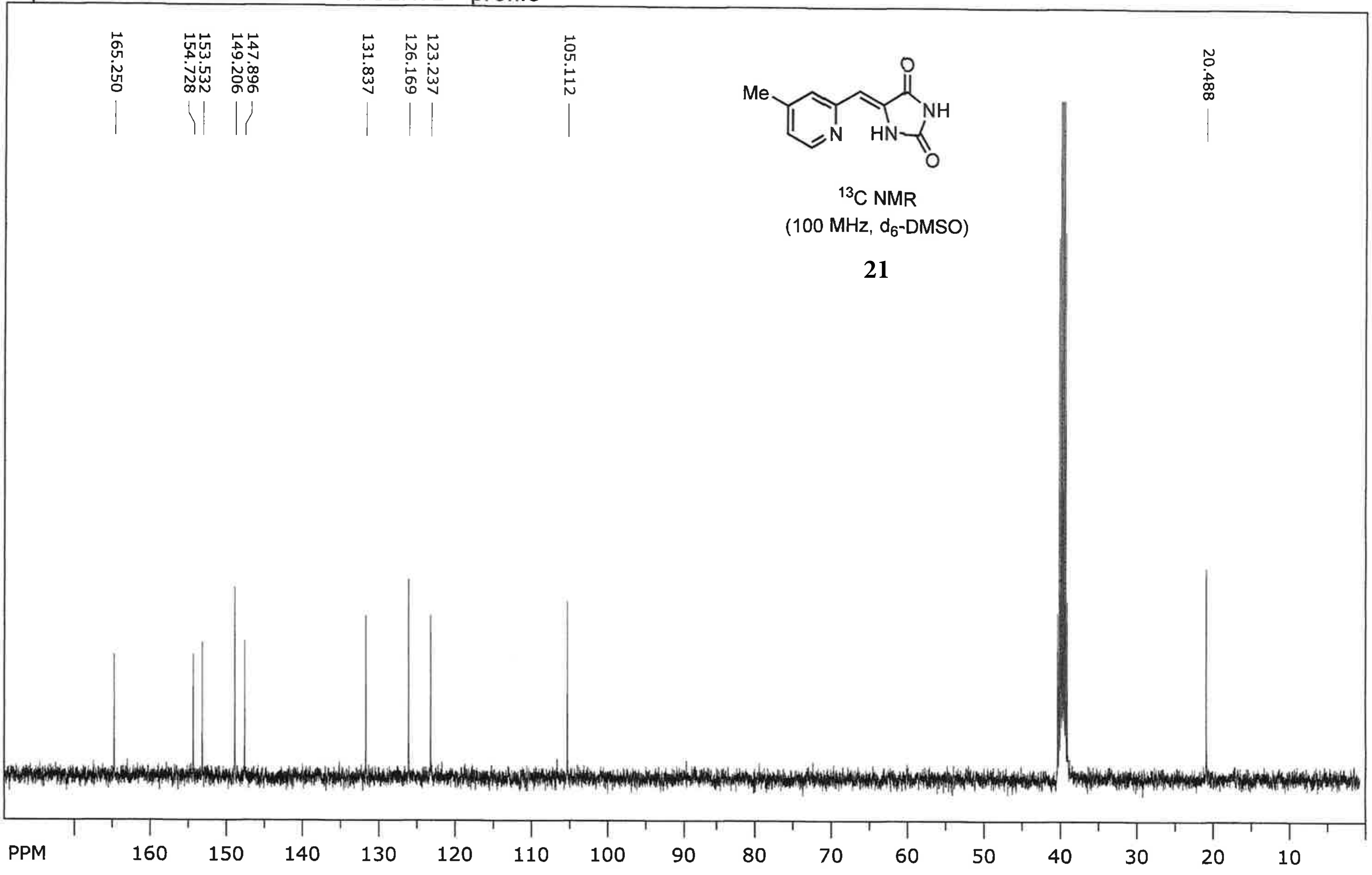

file: ...ectra\WD spectra\WD342_13C.fid\fid block# 1 expt: "s2pul"
transmitter freq.: 100.523180 MHz
time domain size: 65536 points
width: 25000.00 Hz = 248.6989 ppm = 0.381470 Hz/pt
number of scans: 512

freq. of 0 ppm: 100.512160 MHz
processed size: 65536 complex points
LB: 1.500 GF: 0.0000
Hz/cm: 723.333 ppm/cm: 7.19569

SpinWorks 4: JC_SS_1

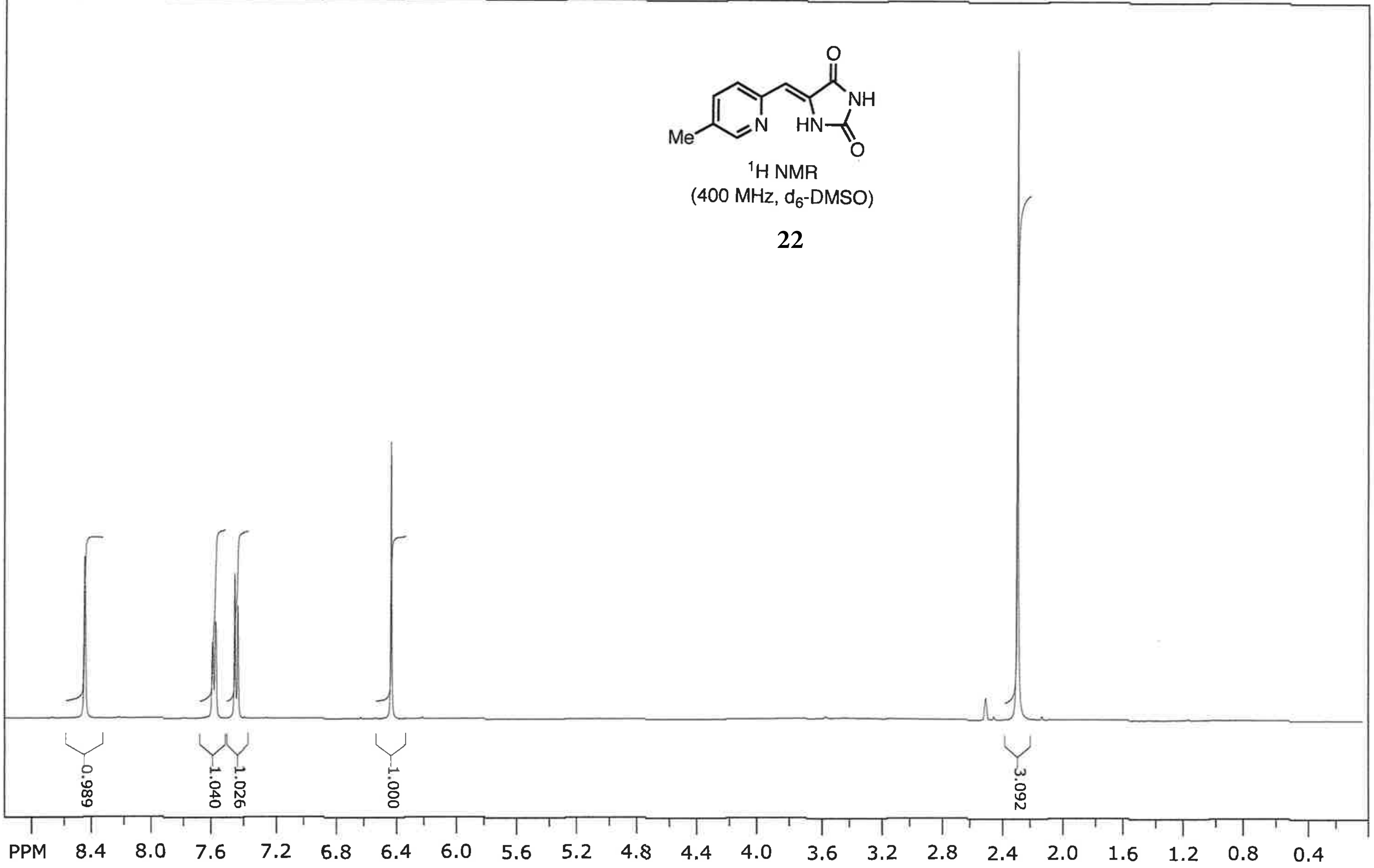

^{1}H NMR
(400 MHz, d_6-DMSO)

22

file: ...son\WD348_1H_DMSO_20240118.fid\fid block# 1 expt: "s2pul"
transmitter freq.: 399.732139 MHz
time domain size: 32768 points
width: 6410.26 Hz = 16.0364 ppm = 0.195626 Hz/pt
number of scans: 16

freq. of 0 ppm: 399.729733 MHz
processed size: 32768 complex points
LB: 0.500 GF: 0.0000
Hz/cm: 143.875 ppm/cm: 0.35993

SpinWorks 4: JC_SS_1

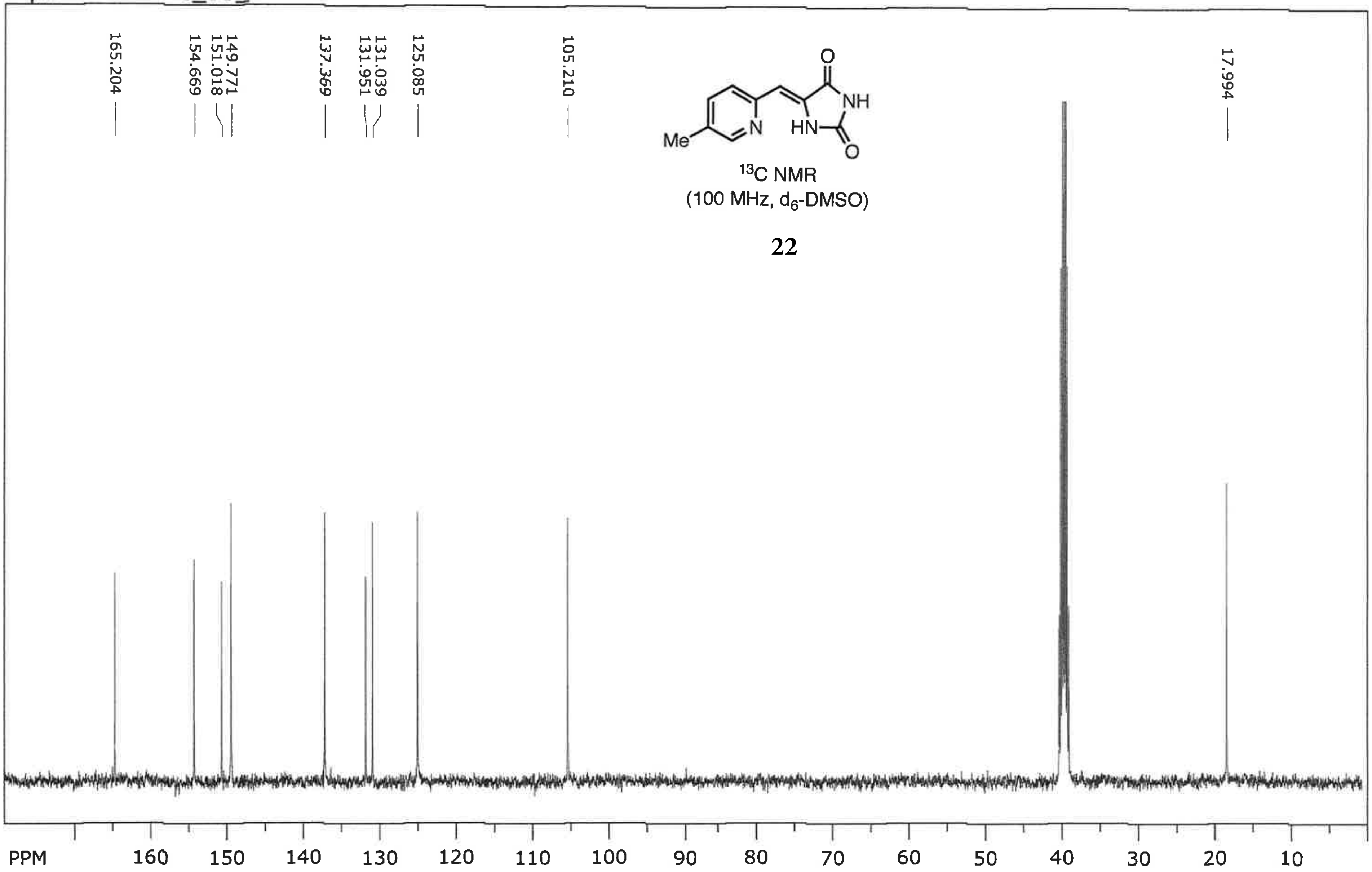

file: ...on\WD348_13C_DMSO_20240118.fid\fid block# 1 expt: "s2pul"
transmitter freq.: 100.523180 MHz
time domain size: 65536 points
width: 25000.00 Hz = 248.6989 ppm = 0.381470 Hz/pt
number of scans: 2048

freq. of 0 ppm: 100.512160 MHz
processed size: 65536 complex points
LB: 2.500 GF: 0.0000
Hz/cm: 723.333 ppm/cm: 7.19569

SpinWorks 4: STANDARD 1H OBSERVE

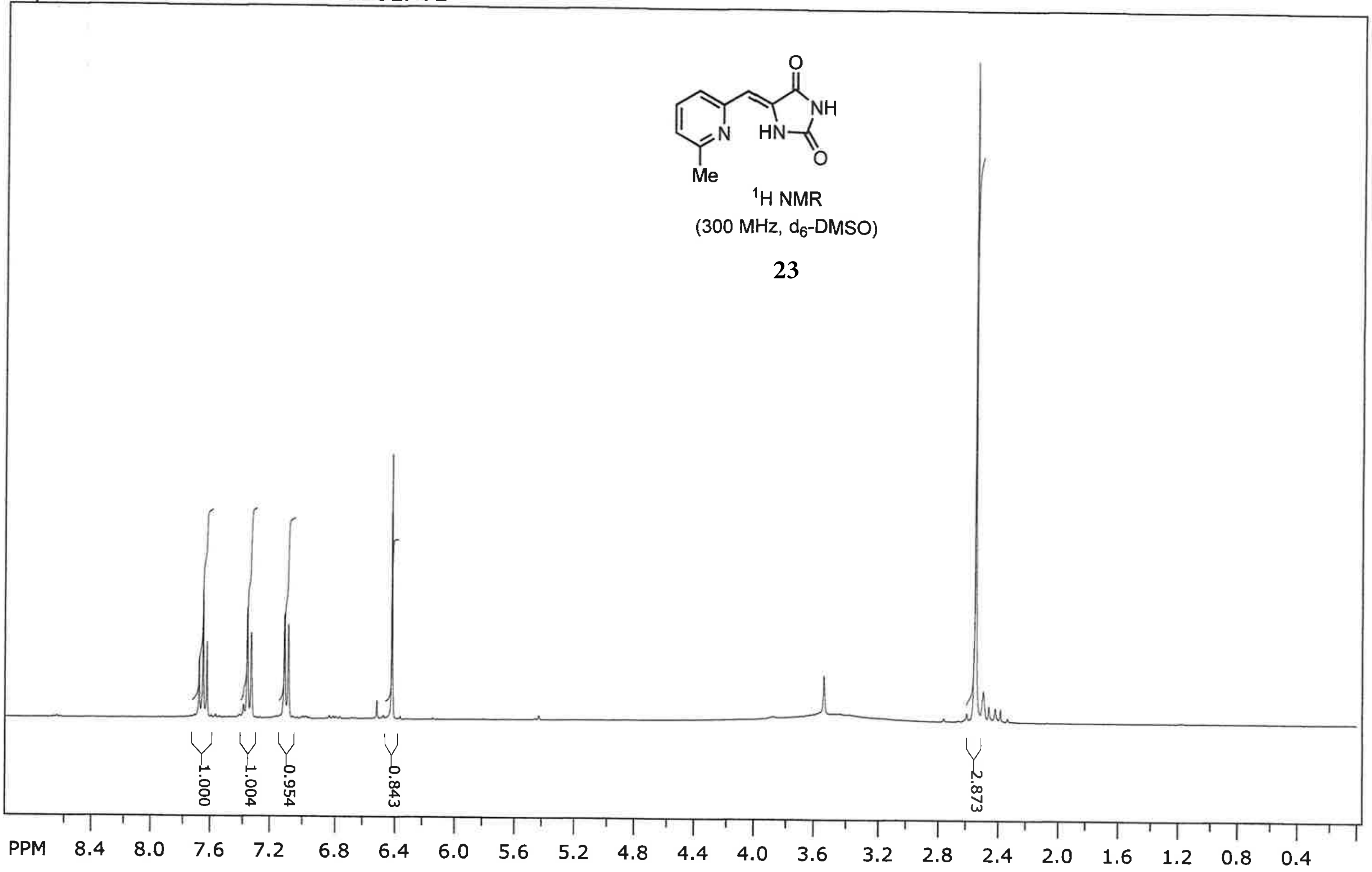

file: ...pectra\WD spectra\WD329_1H.fid\fid block# 1 expt: "s2pul"
transmitter freq.: 300.134434 MHz
time domain size: 19192 points
width: 4803.07 Hz = 16.0031 ppm = 0.250264 Hz/pt
number of scans: 8

freq. of 0 ppm: 300.132634 MHz
processed size: 32768 complex points
LB: 0.500 GF: 0.0000
Hz/cm: 108.016 ppm/cm: 0.35989

SpinWorks 4: JC_SS_1

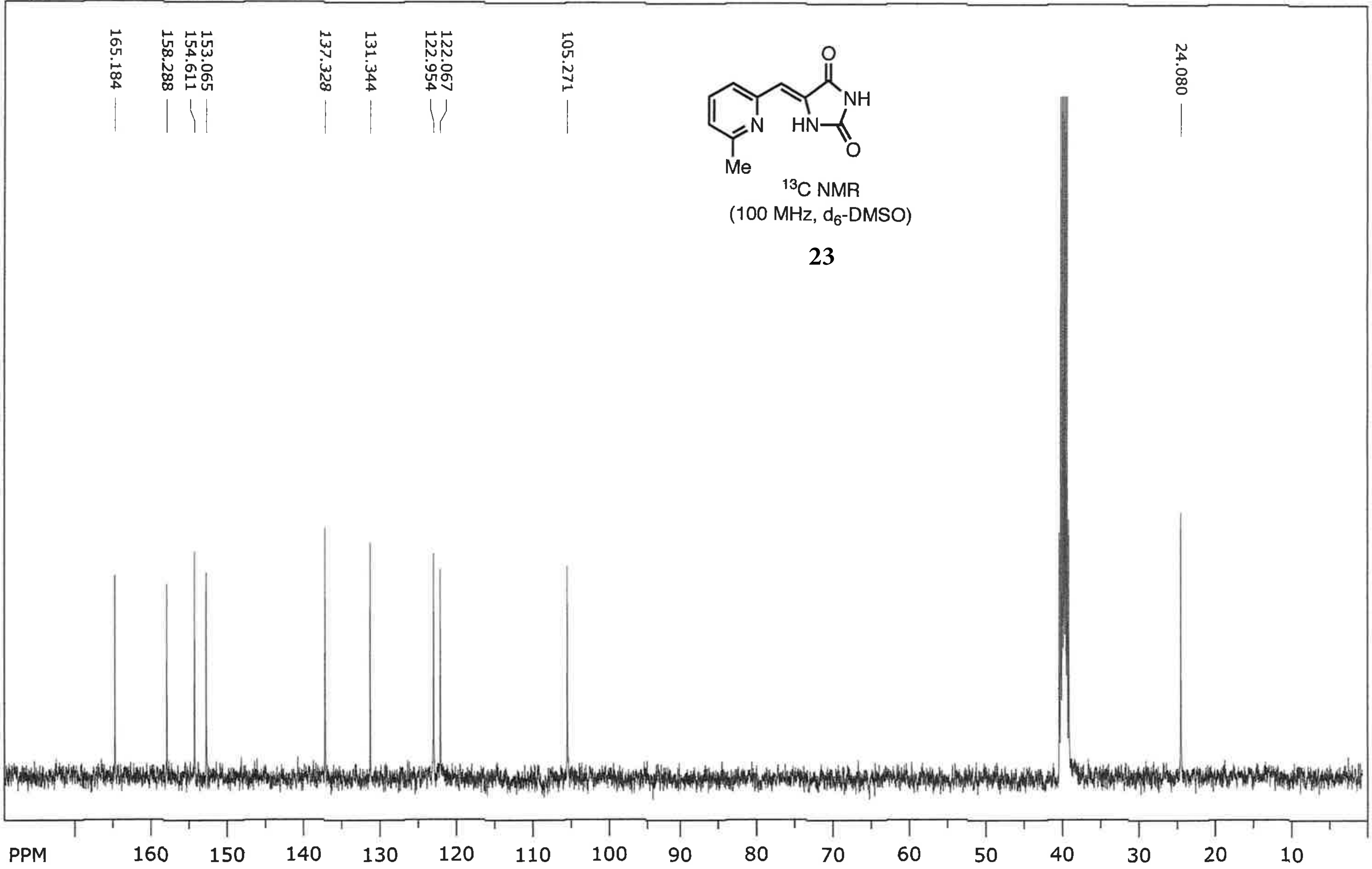

^{13}C NMR
(100 MHz, d_6-DMSO)

23

file: ...on\WD329_13C_DMSO_20240116.fid\fid block# 1 expt: "s2pul"
transmitter freq.: 100.523180 MHz
time domain size: 65536 points
width: 25000.00 Hz = 248.6989 ppm = 0.381470 Hz/pt
number of scans: 1088

freq. of 0 ppm: 100.512161 MHz
processed size: 65536 complex points
LB: 2.500 GF: 0.0000
Hz/cm: 723.333 ppm/cm: 7.19569

SpinWorks 4: 9_28_20_EAW995_TopLayer

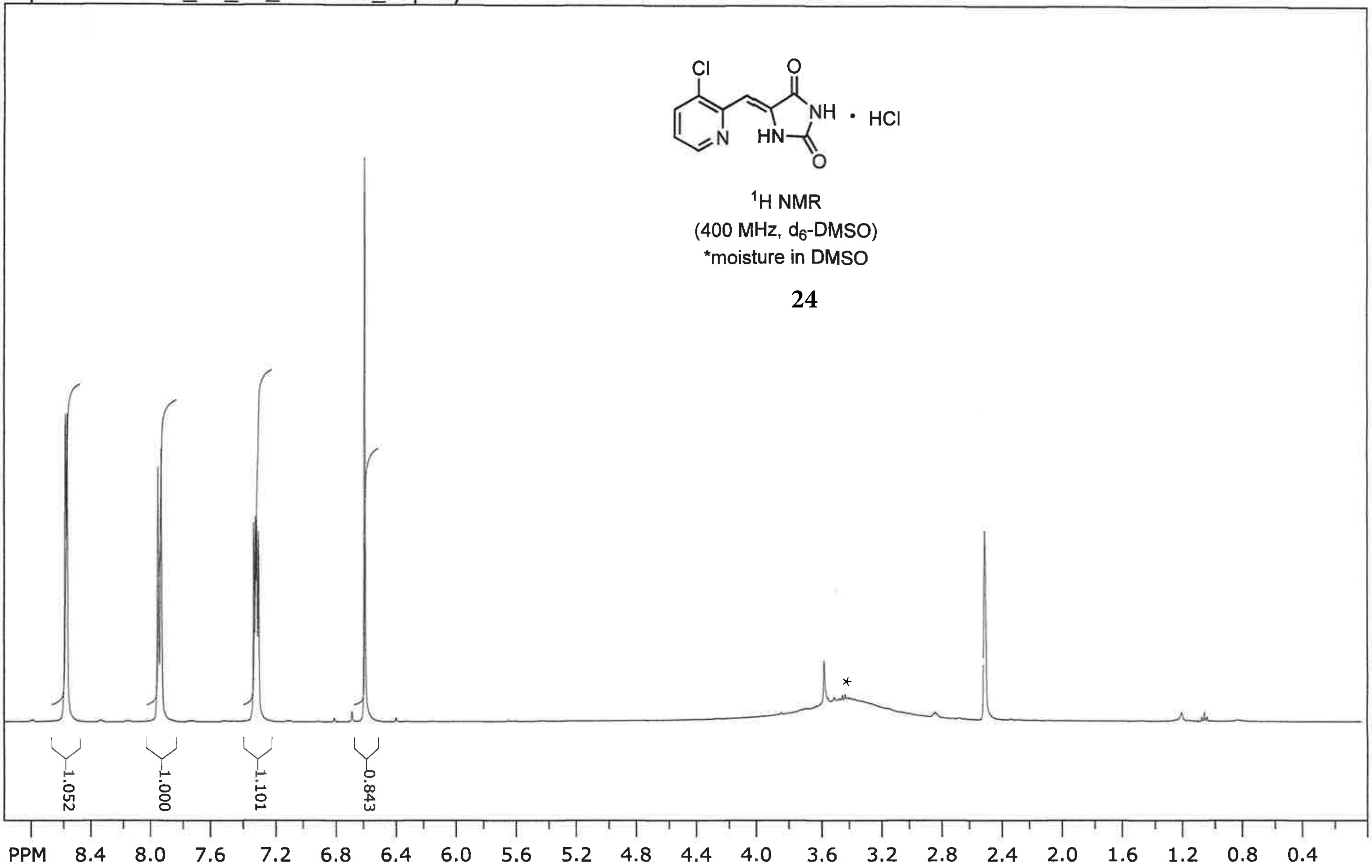

file: ...spectra\WD340-349\WD346-1H.fid\fid block# 1 expt: "s2pul"
transmitter freq.: 399.732139 MHz
time domain size: 32768 points
width: 6410.26 Hz = 16.0364 ppm = 0.195626 Hz/pt
number of scans: 8

freq. of 0 ppm: 399.729734 MHz
processed size: 32768 complex points
LB: 0.510 GF: 0.0000
Hz/cm: 143.875 ppm/cm: 0.35993

SpinWorks 4: new experiment

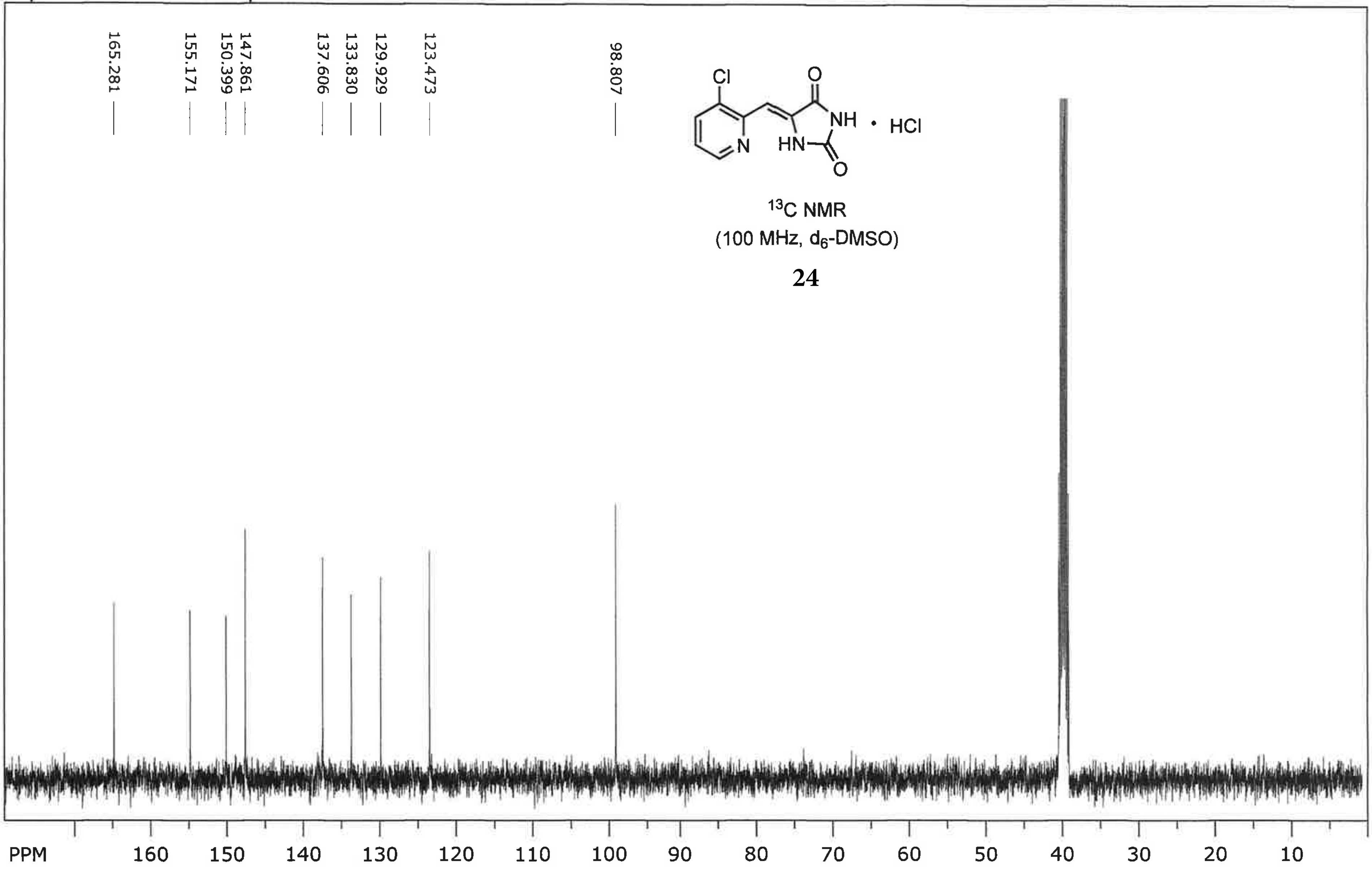

file: ...pectra\WD340-349\WD346-13C.fid\fid block# 1 expt: "s2pul"
transmitter freq.: 100.523180 MHz
time domain size: 65536 points
width: 25000.00 Hz = 248.6989 ppm = 0.381470 Hz/pt
number of scans: 512

freq. of 0 ppm: 100.512163 MHz
processed size: 65536 complex points
LB: 1.500 GF: 0.0000
Hz/cm: 723.333 ppm/cm: 7.19569

SpinWorks 4: 9_28_20_EAW995_TopLayer

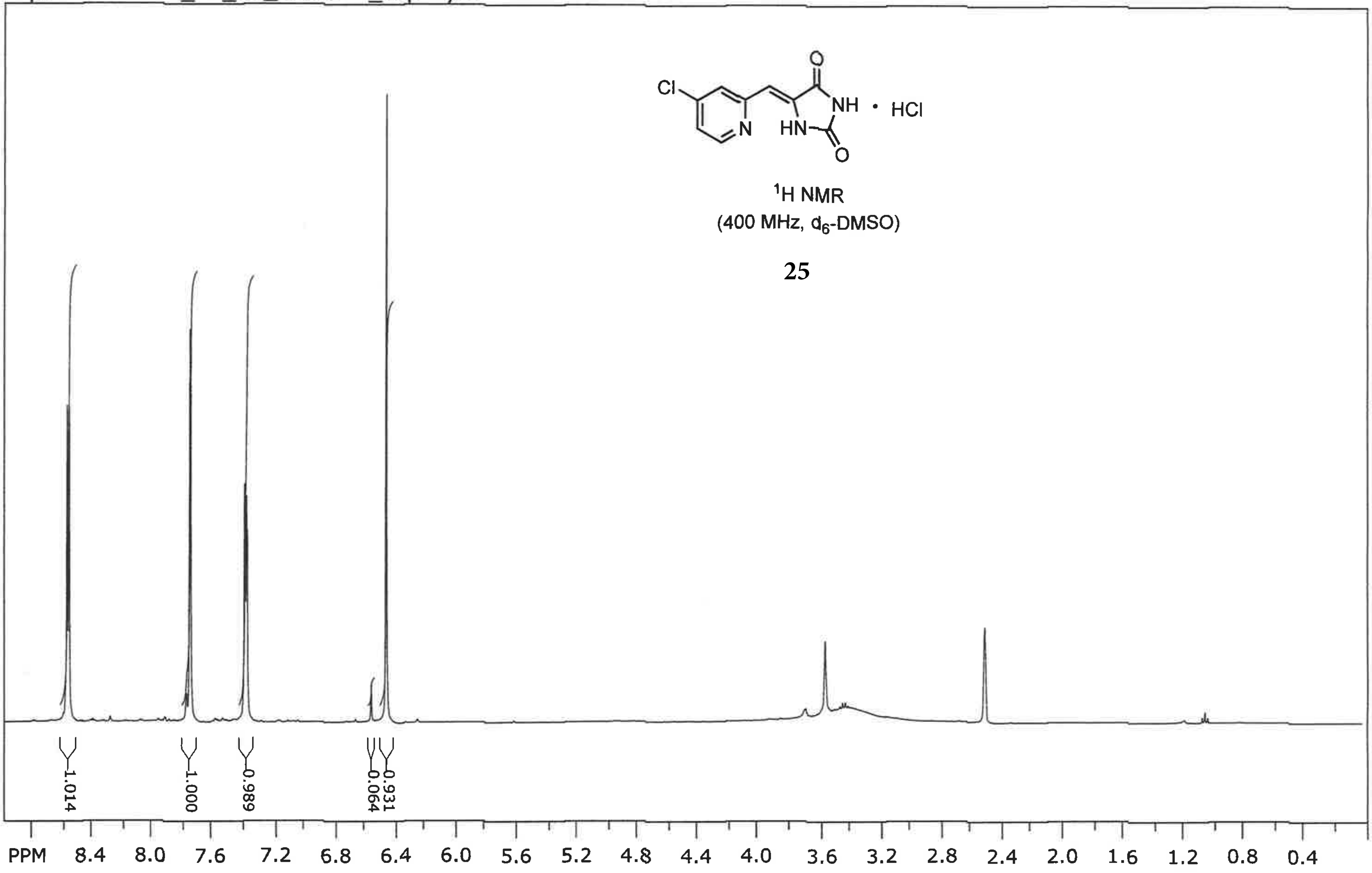

file: ...spectra\WD340-349\WD347-1H.fid\fid block# 1 expt: "s2pul"
transmitter freq.: 399.732139 MHz
time domain size: 32768 points
width: 6410.26 Hz = 16.0364 ppm = 0.195626 Hz/pt
number of scans: 8

freq. of 0 ppm: 399.729734 MHz
processed size: 32768 complex points
LB: 0.610 GF: 0.0000
Hz/cm: 143.875 ppm/cm: 0.35993

SpinWorks 4: new experiment

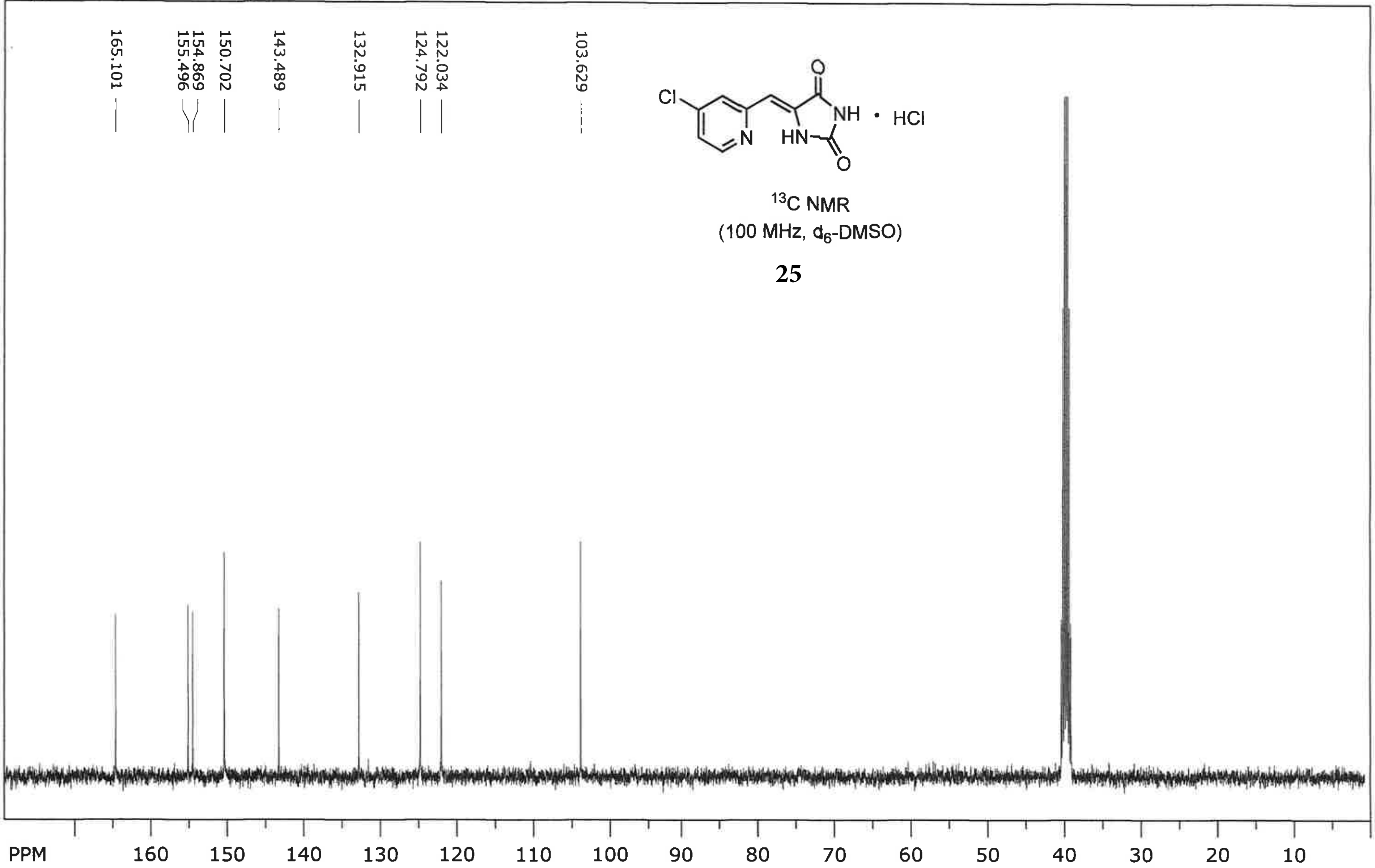

^{13}C NMR
(100 MHz, d_6-DMSO)

25

file: ...pectra\WD340-349\WD347-13C.fid\fid block# 1 expt: "s2pul"
transmitter freq.: 100.523180 MHz
time domain size: 65536 points
width: 25000.00 Hz = 248.6989 ppm = 0.381470 Hz/pt
number of scans: 512

freq. of 0 ppm: 100.512162 MHz
processed size: 65536 complex points
LB: 1.500 GF: 0.0000
Hz/cm: 723.333 ppm/cm: 7.19569

SpinWorks 4: 12_10_20_Diester_carbon

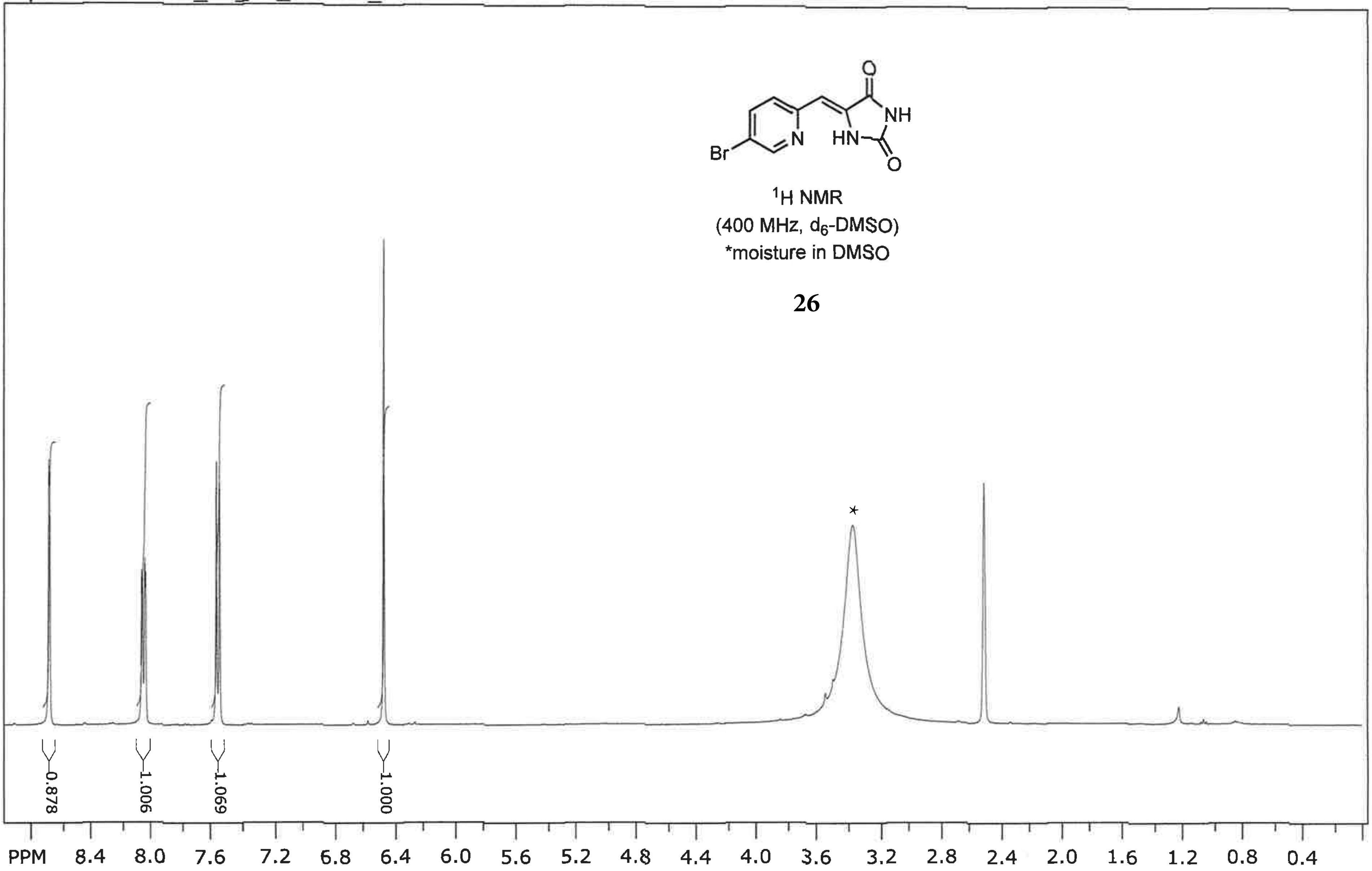

^{1}H NMR
(400 MHz, d_6-DMSO)
*moisture in DMSO

26

file: ...spectra\WD330-339\WD333-H1.fid\fid block# 1 expt: "s2pul"
transmitter freq.: 399.732139 MHz
time domain size: 32768 points
width: 6410.26 Hz = 16.0364 ppm = 0.195626 Hz/pt
number of scans: 8

freq. of 0 ppm: 399.729733 MHz
processed size: 32768 complex points
LB: 0.610 GF: 0.0000
Hz/cm: 144.017 ppm/cm: 0.36028

SpinWorks 4: wefarv

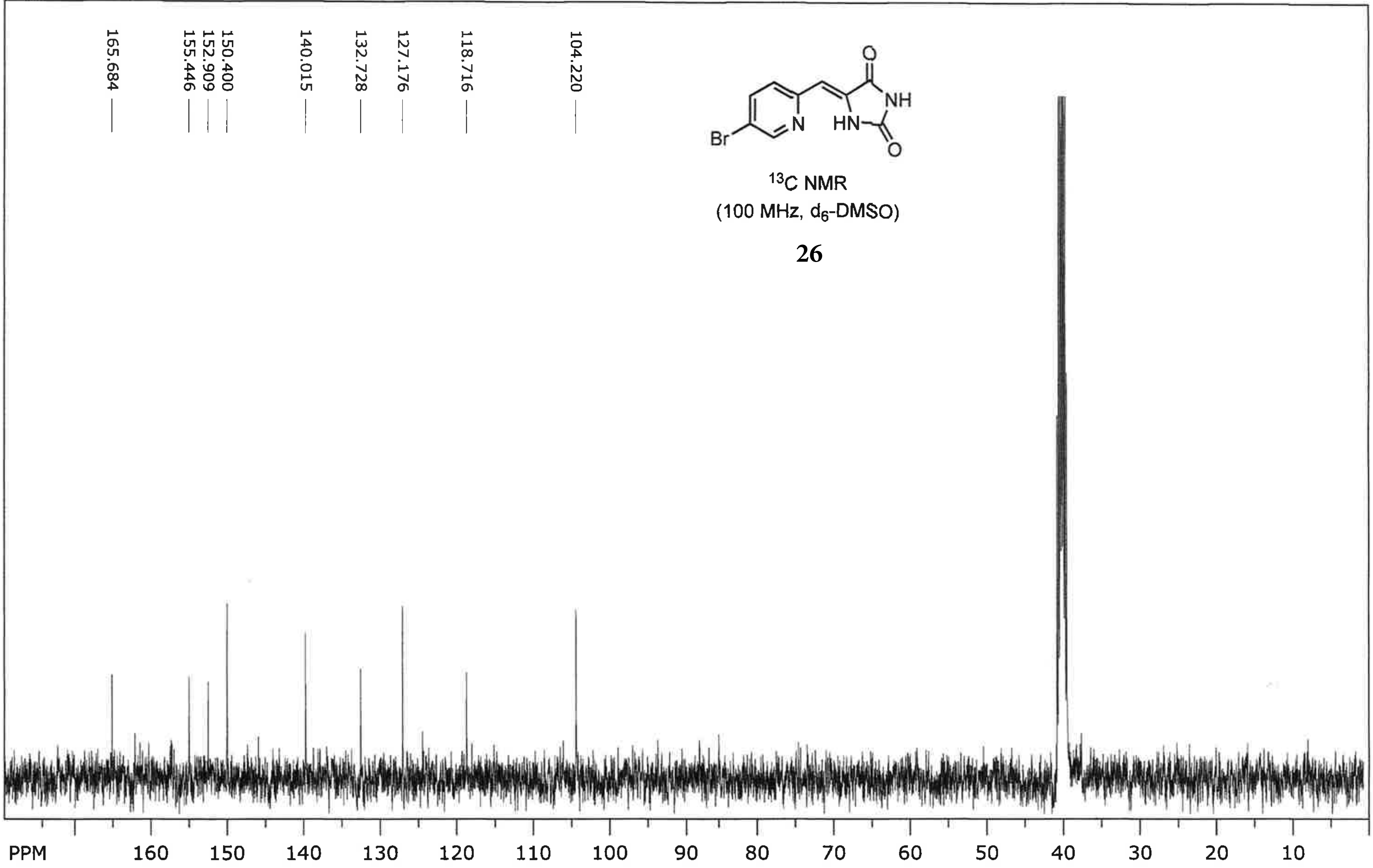

file: ...pectra\WD330-339\WD333-C13.fid\fid block# 1 expt: "s2pul"
transmitter freq.: 100.523180 MHz
time domain size: 65536 points
width: 25000.00 Hz = 248.6989 ppm = 0.381470 Hz/pt
number of scans: 256

freq. of 0 ppm: 100.512123 MHz
processed size: 65536 complex points
LB: 2.500 GF: 0.0000
Hz/cm: 723.889 ppm/cm: 7.20121

SpinWorks 4: STANDARD 1H OBSERVE - profile

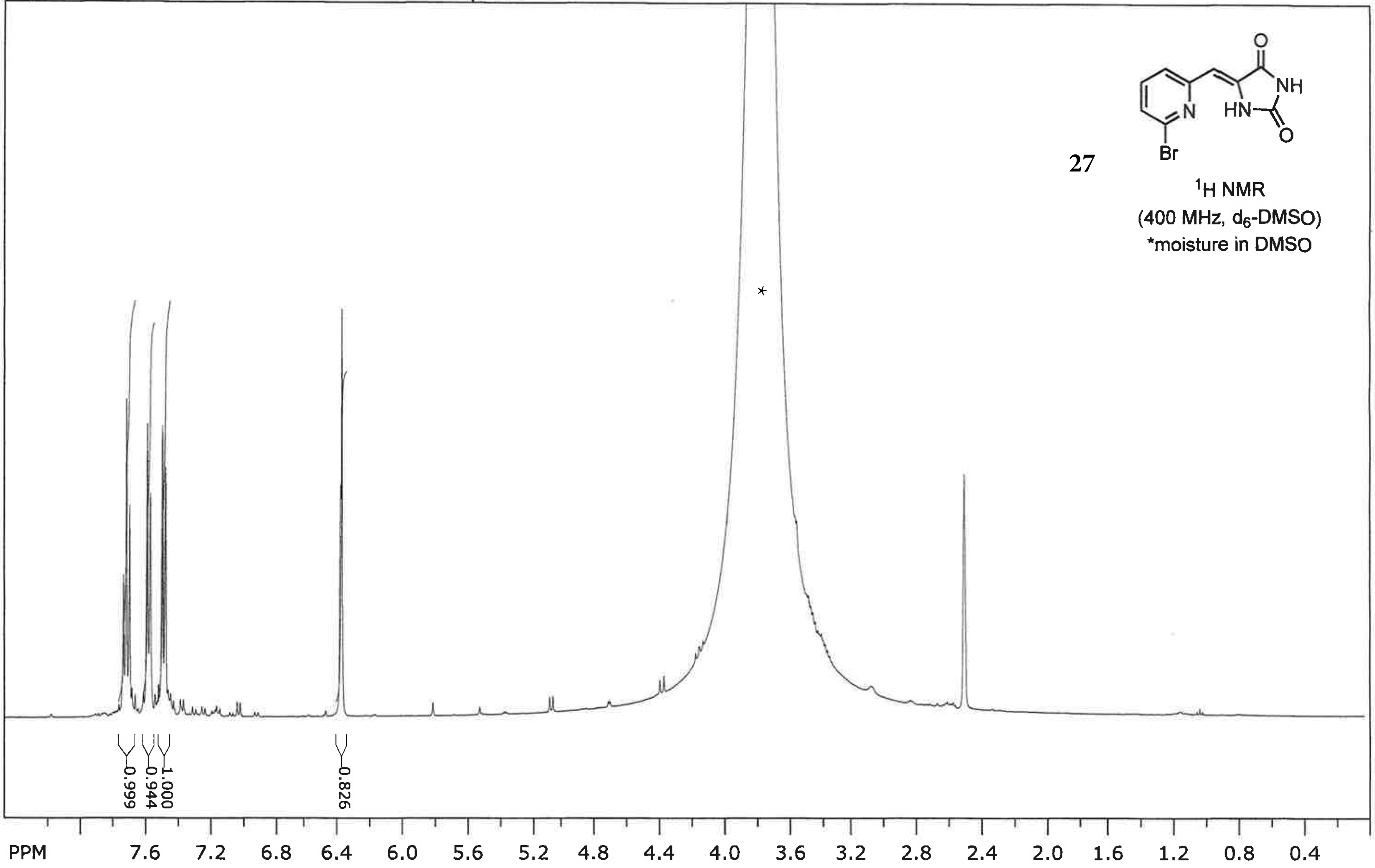

file: ...pectra\WD spectra\WD326_1H.fid\fid block# 1 expt: "s2pul"
transmitter freq.: 399.732139 MHz
time domain size: 32768 points
width: 6410.26 Hz = 16.0364 ppm = 0.195626 Hz/pt
number of scans: 8

freq. of 0 ppm: 399.729734 MHz
processed size: 32768 complex points
LB: 0.506 GF: 0.0000
Hz/cm: 135.897 ppm/cm: 0.33997

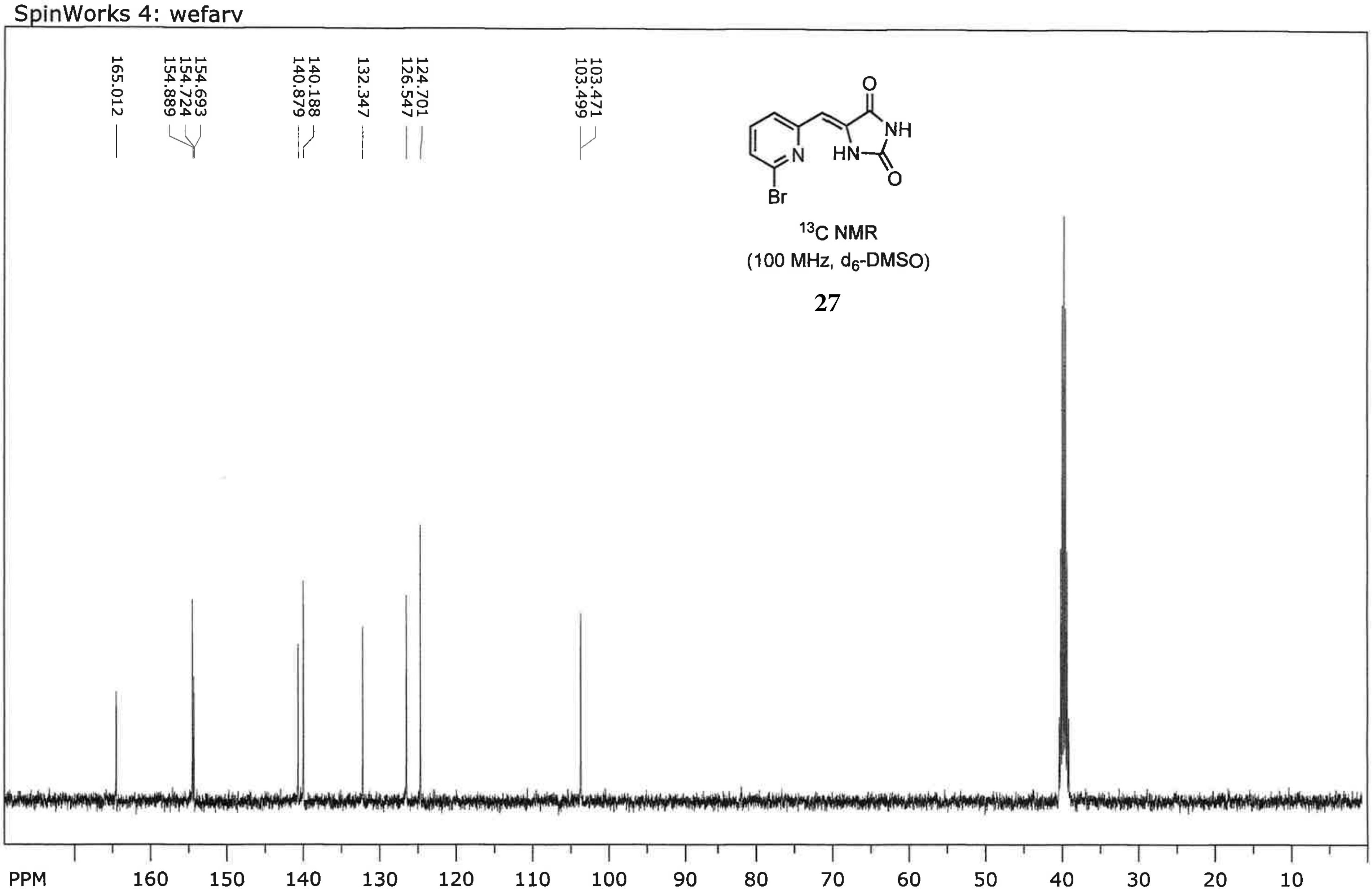

file: ...pectra\WD320-329\WD327-C13.fid\fid block# 1 expt: "s2pul"
transmitter freq.: 100.523180 MHz
time domain size: 65536 points
width: 25000.00 Hz = 248.6989 ppm = 0.381470 Hz/pt
number of scans: 256

freq. of 0 ppm: 100.512160 MHz
processed size: 65536 complex points
LB: 1.500 GF: 0.0000
Hz/cm: 723.333 ppm/cm: 7.19569

SpinWorks 4: rr

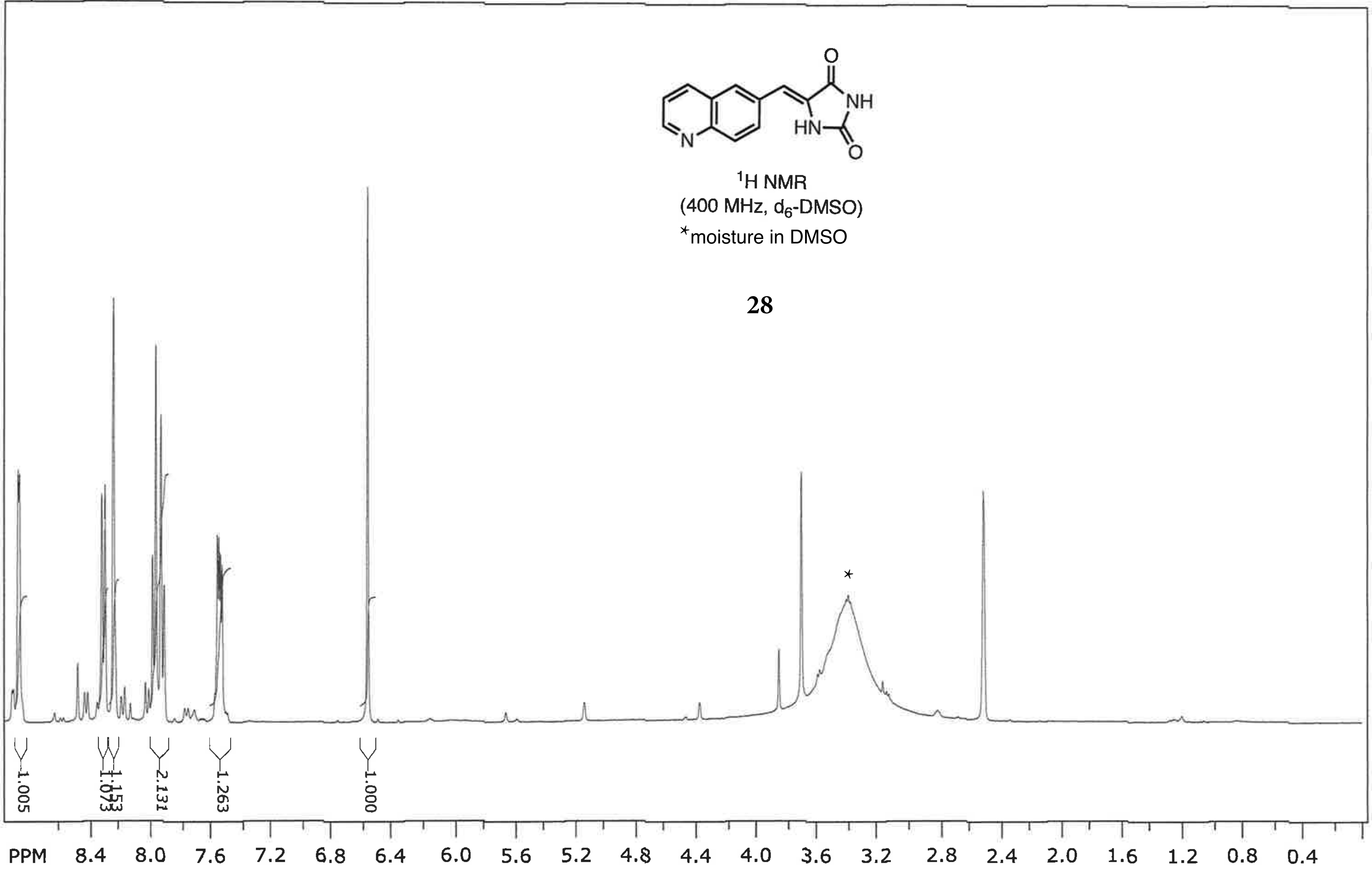

file: ...pectra\KG01-10\KG10-proton.fid\fid block# 1 expt: "s2pul"
transmitter freq.: 399.732139 MHz
time domain size: 32768 points
width: 6410.26 Hz = 16.0364 ppm = 0.195626 Hz/pt
number of scans: 8

freq. of 0 ppm: 399.729733 MHz
processed size: 32768 complex points
LB: 0.500 GF: 0.0000
Hz/cm: 143.732 ppm/cm: 0.35957

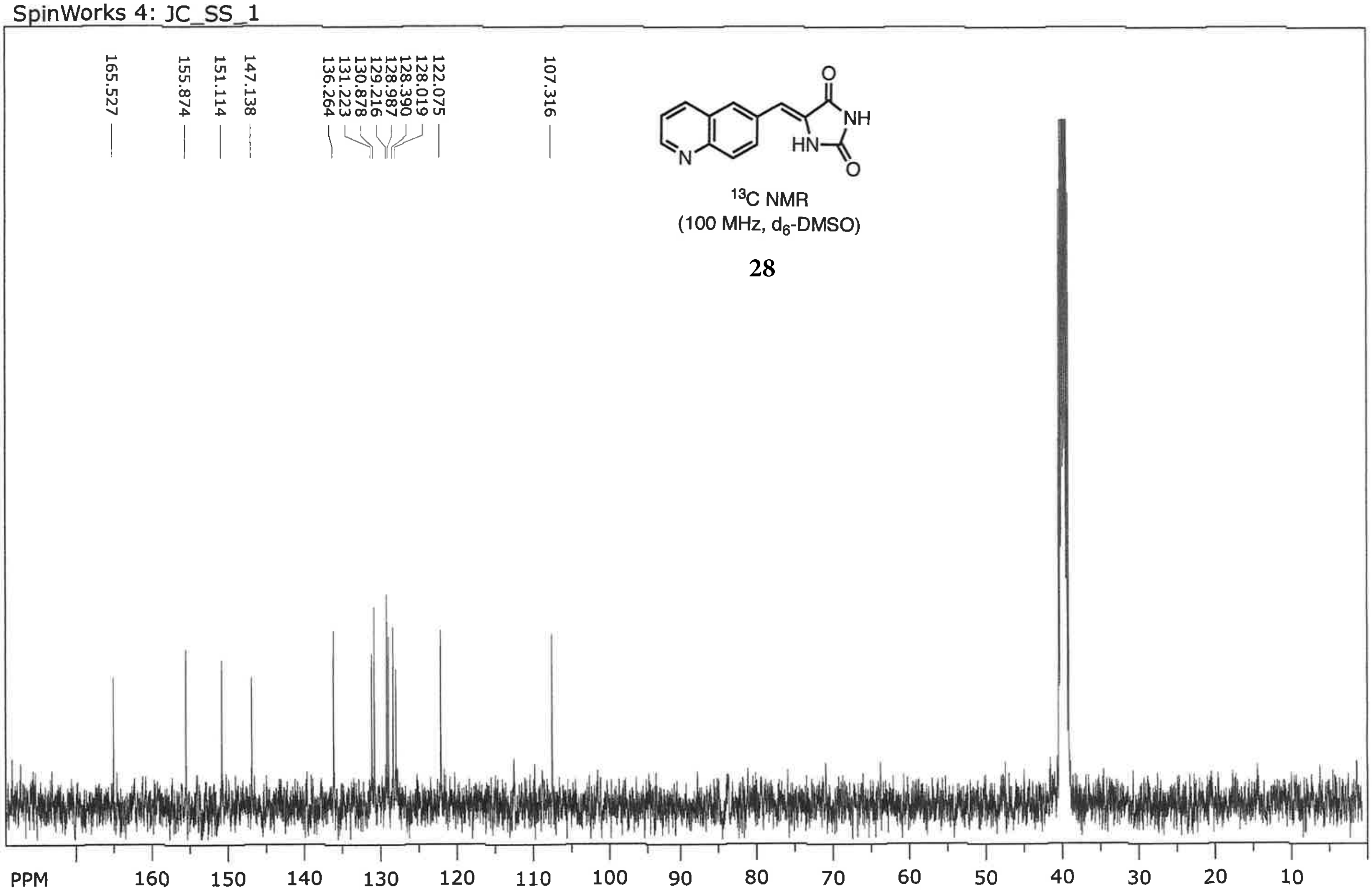

file: ...son\KG10_13C_DMSO_20240118.fid\fid block# 1 expt: "s2pul"
transmitter freq.: 100.523180 MHz
time domain size: 65536 points
width: 25000.00 Hz = 248.6989 ppm = 0.381470 Hz/pt
number of scans: 2048

freq. of 0 ppm: 100.512162 MHz
processed size: 65536 complex points
LB: 2.500 GF: 0.0000
Hz/cm: 723.333 ppm/cm: 7.19569

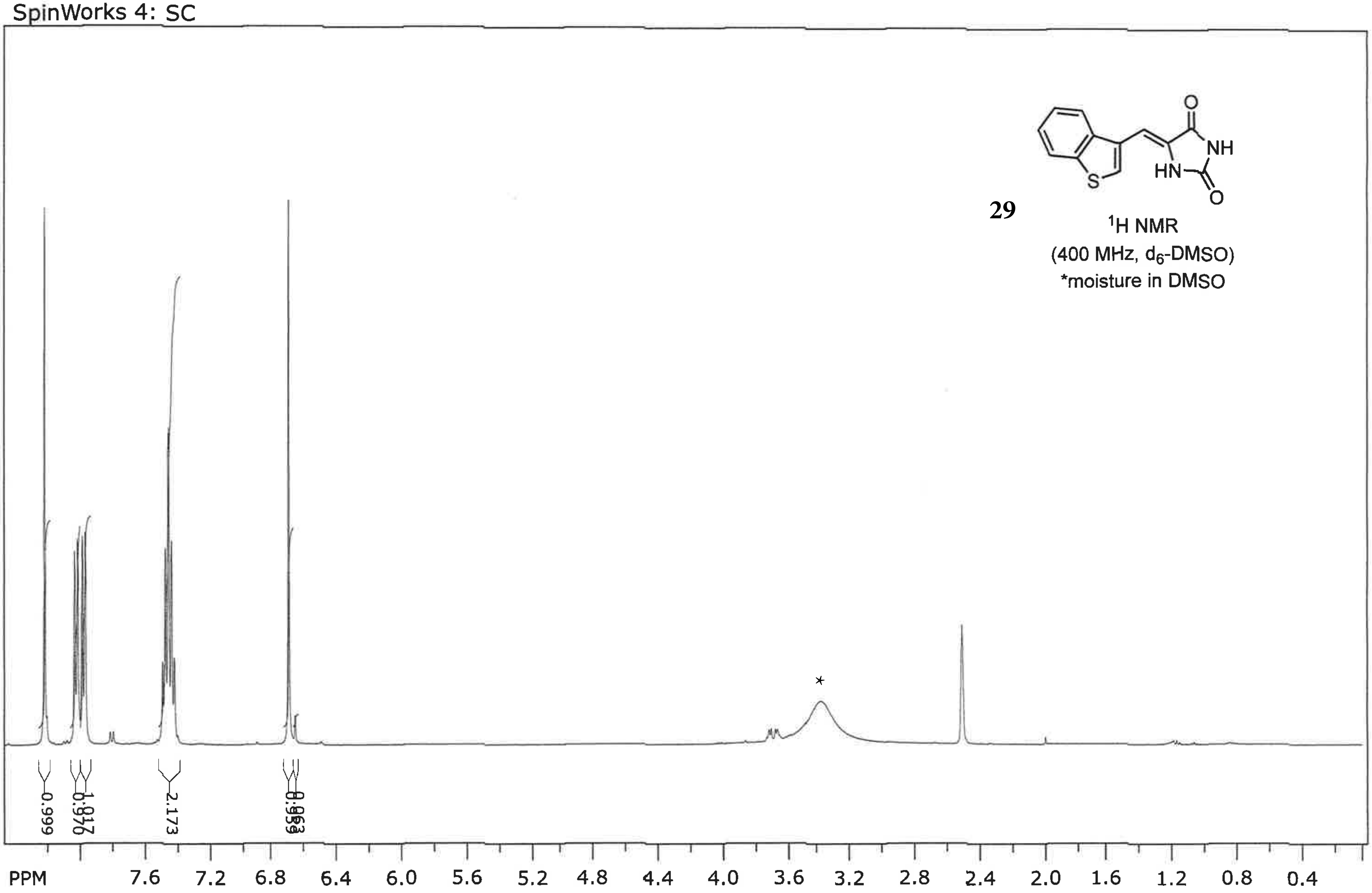

file: ...pectra\KG11-20\KG18-proton.fid\fid block# 1 expt: "s2pul"
transmitter freq.: 399.732139 MHz
time domain size: 32768 points
width: 6410.26 Hz = 16.0364 ppm = 0.195626 Hz/pt
number of scans: 8

freq. of 0 ppm: 399.729735 MHz
processed size: 32768 complex points
LB: 0.500 GF: 0.0000
Hz/cm: 135.897 ppm/cm: 0.33997

SpinWorks 4: EAW1050_carbon

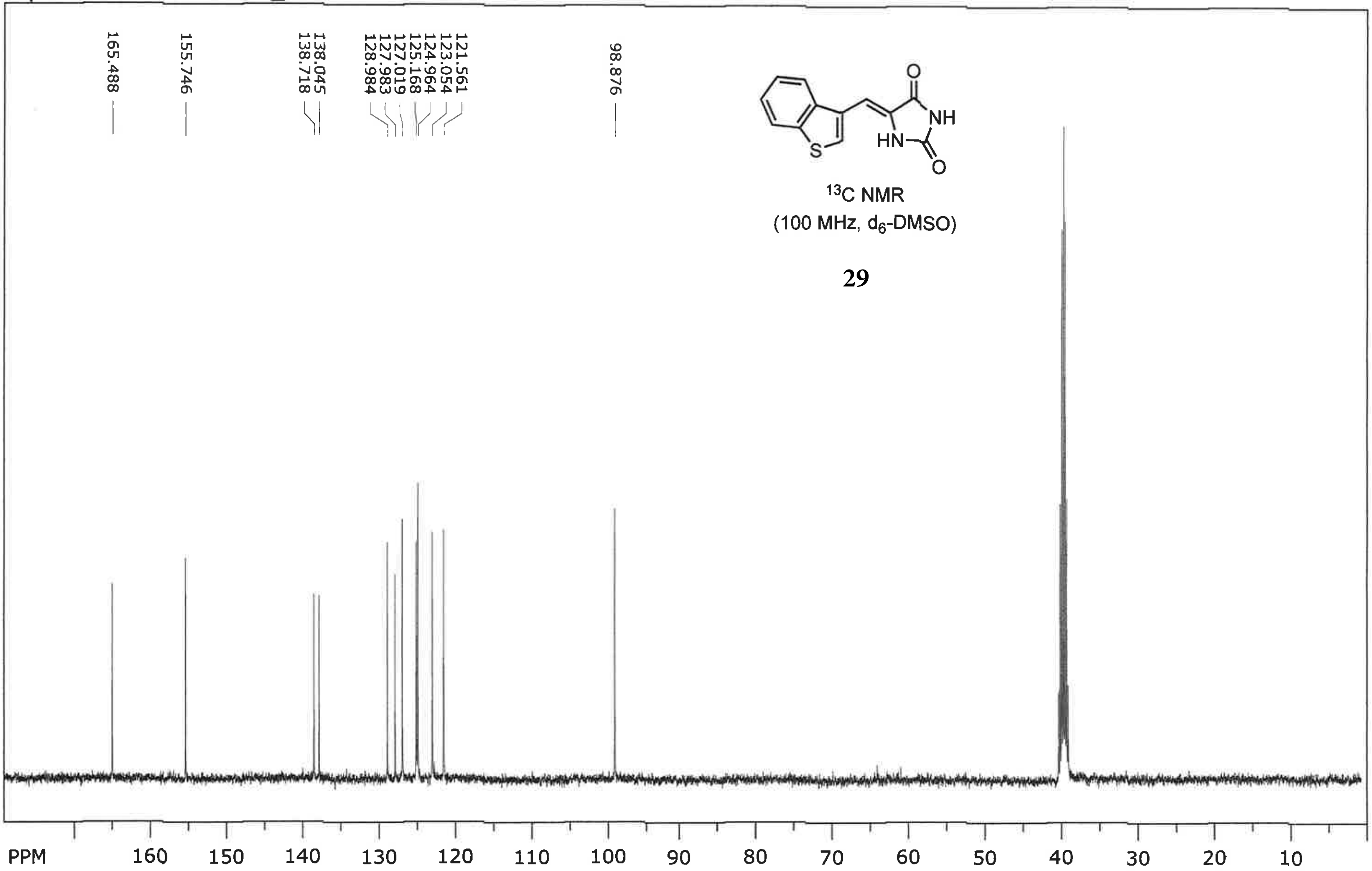

file: ...ldsonw\Desktop\KG18-carbon.fid\fid block# 1 expt: "s2pul"
transmitter freq.: 100.523180 MHz
time domain size: 65536 points
width: 25000.00 Hz = 248.6989 ppm = 0.381470 Hz/pt
number of scans: 256

freq. of 0 ppm: 100.512160 MHz
processed size: 65536 complex points
LB: 1.500 GF: 0.0000
Hz/cm: 723.333 ppm/cm: 7.19569
